# Association of Oral Anticoagulation With Stroke in Atrial Fibrillation or Heart Failure: A Comparative Meta-Analysis

**DOI:** 10.1161/STROKEAHA.120.033910

**Published:** 2021-07-20

**Authors:** Catriona Reddin, Conor Judge, Elaine Loughlin, Robert Murphy, Maria Costello, Alberto Alvarez, John Ferguson, Andrew Smyth, Michelle Canavan, Martin J. O’Donnell

**Affiliations:** 1 HRB-Clinical Research Facility (C.R., C.J., E.L., R.M., M. Costello, A.A., J.F., A.S., M. Canavan, M.J.O.), National University of Ireland Galway.; 2 Translational Medical Device Laboratory (C.J.), National University of Ireland Galway.; 3 Department of Geriatric and Stroke Medicine, Galway University Hospital, Newcastle Road, Ireland (C.R., C.J., E.L., R.M., M. Costello, A.S., M. Canavan, M.J.O.).; 4 Wellcome Trust – HRB, Irish Clinical Academic Training (C.J.).

**Keywords:** anticoagulants, atrial fibrillation, heart failure, odds ratio, uncertainty

## Abstract

Atrial fibrillation and heart failure with reduced ejection fraction (HFrEF) are common sources of cardioembolism. While oral anticoagulation is strongly recommended for atrial fibrillation, there are marked variations in guideline recommendations for HFrEF due to uncertainty about net clinical benefit. This systematic review and meta-analysis evaluates the comparative association of oral anticoagulation with stroke and other cardiovascular risk in populations with atrial fibrillation or HFrEF in sinus rhythm and identify factors mediating different estimates of net clinical benefit.

**Methods:**

PubMed and Embase were searched from database inception to November 20, 2019 for randomized clinical trials comparing oral anticoagulation to control. A random-effects meta-analysis was used to estimate a pooled treatment-effect overall and within atrial fibrillation and HFrEF trials. Differences in treatment effect were assessed by estimating I^2^ among all trials and testing the between-trial-population *P*-interaction. The primary outcome measure was all stroke. Secondary outcome measures were ischemic stroke, hemorrhagic stroke, mortality, myocardial infarction, and major hemorrhage.

**Results:**

Twenty-one trials were eligible for inclusion, 15 (n=19 332) in atrial fibrillation (mean follow-up: 23.1 months), and 6 (n=9866) in HFrEF (mean follow-up: 23.9 months). There were differences in primary outcomes between trial populations, with all-cause mortality included for 95.2% of HFrEF trial population versus 0.38% for atrial fibrillation. Mortality was higher in controls groups of HFrEF populations (19.0% versus 9.6%) but rates of stroke lower (3.1% versus 7.0%) compared with atrial fibrillation. The association of oral anticoagulation with all stroke was consistent for atrial fibrillation (odds ratio, 0.51 [95% CI, 0.42–0.63]) and HFrEF (odds ratio, 0.61 [95% CI, 0.47–0.79]; I^2^=12.4%; *P* interaction=0.31). There were no statistically significant differences in the association of oral anticoagulation with cardiovascular events, mortality or bleeding between populations.

**Conclusions:**

The relative association of oral anticoagulation with stroke risk, and other cardiovascular outcomes, is similar for patients with atrial fibrillation and HFrEF. Differences in the primary outcomes employed by trials in HFrEF, compared with atrial fibrillation, may have contributed to differing conclusions of the relative efficacy of oral anticoagulation.

## Abstract 2

Supplemental Digital Content is available in the text.

Atrial fibrillation and heart failure with reduced ejection fraction (HFrEF) are common causes of cardioembolic stroke. Oral anticoagulation is strongly recommended (Grade 1A) for patients with atrial fibrillation to reduce the risk of ischemic stroke.^1–3^ In contrast, clinical trials in patients with HFrEF in sinus rhythm have not reported superiority of oral anticoagulation versus antiplatelet therapy or control for cardiovascular prevention,^4–6^ and guideline recommendations are inconsistent.^7,8^ For example, the Heart Failure Society of America recommends anticoagulation with warfarin, with an international normalized ratio (INR) target of 2 to 3 for patients with HFrEF and a history of thromboembolism.^7,9^ Conversely, the American Heart Association make a level B recommendation against anticoagulation in heart failure in the absence of atrial fibrillation.^8^ Several factors may explain the apparent difference in the efficacy, or interpretation of efficacy from clinical trials, of oral anticoagulation in atrial fibrillation compared with HFrEF in sinus rhythm. First, the mechanism of ischemic stroke may differ between populations, and patients with HFrEF may have a higher prevalence of competing stroke etiologies, for which oral anticoagulation is not superior to antiplatelet therapy (eg, small vessel disease). Second, the risks associated with oral anticoagulant therapy may differ in patients with HFrEF than in patients with atrial fibrillation, for example, the risk of intracerebral hemorrhage, which may offset benefits in the reduction of ischemic stroke.^10,11^ Third, differences in the primary outcome measures between trials of patients with HFrEF (which mostly used composite outcomes, including all-cause mortality) and trials of patients with atrial fibrillation (which mostly used all stroke) may account for the observed difference in efficacy of oral anticoagulation. In addition, there may be differences in the relative contribution of stroke to a composite outcome between populations.

While prior meta-analyses have examined the effectiveness of oral anticoagulation in patients with atrial fibrillation or HFrEF individually, our meta-analysis specifically evaluates the comparative association of oral anticoagulation with incidence of stroke, other cardiovascular events and mortality in populations with atrial fibrillation compared with populations with HFrEF in sinus rhythm.

## Methods

We performed a systematic review and meta-analysis, adhering to the Cochrane Collaboration Guidelines and reported our findings according to the Preferred Reporting Items for Systematic Reviews and Meta-Analyses Guidelines.^12,13^ The meta-analysis was registered with the International Prospective Register of Systematic Reviews (REGISTRATION: URL: https://www.crd.york.ac.uk/prospero/; PROSPERO identifier: CRD42020153013). The data that support the findings of this study are available from the corresponding author upon reasonable request.

### Data Sources and Search Strategy

We systematically searched PubMed and Embase databases from database inception to November 20, 2019. The search terms included are detailed in Methods I in the Data Supplement. The search strategy was peer-reviewed by an information specialist. Following removal of duplicates, titles and abstracts were screened by 2 reviewers (C.R. and E.L.) using the Rayann web application.^14^ The reference lists of included trials and other published meta-analyses were also reviewed. Full texts of remaining articles were independently assessed by 2 reviewers (C.R. and E.L.), with eligibility based on predetermined criteria. Disagreements were resolved by consensus, where a resolution was not reached by discussion, a consensus was reached through a third reviewer (C.J.).

### Eligibility Criteria

Studies were considered eligible if they (1) were randomized clinical trials; (2) included adults >18 years; (3) evaluated oral anticoagulation compared with control; and (4) reported stroke events (at least one of the following: all stroke, ischemic stroke, or hemorrhagic stroke). Control was defined as antiplatelet, placebo, or no antithrombotic treatment.

### Data Extraction/Measurements

Data were extracted independently by 2 authors (C.R. and E.L.) using a standardized predetermined data collection form. For each study, we extracted the title, year of publication, oral anticoagulant (including dose/target), antiplatelet (including dose, where applicable), active and control numbers, all stroke, ischemic stroke, hemorrhagic stroke, all-cause mortality, cardiovascular mortality, total myocardial infarction, major hemorrhage, fatal hemorrhage, and original primary outcomes of individual trials. We did not prespecify a definition for stroke or major hemorrhage. Data were compared for inconsistencies and merged into a prefinal dataset, which was checked independently by 2 other reviewers (C.J. and M.C.).

### Outcomes

The primary outcome measure was all stroke. The secondary outcome measures were ischemic stroke, hemorrhagic stroke, all-cause mortality, cardiovascular mortality, total myocardial infarction, major hemorrhage, fatal hemorrhage, and original primary outcomes of individual trials. The definition of original primary outcomes of individual trials and major hemorrhage differed among trials (Table; Table I in the Data Supplement).

**Table.**
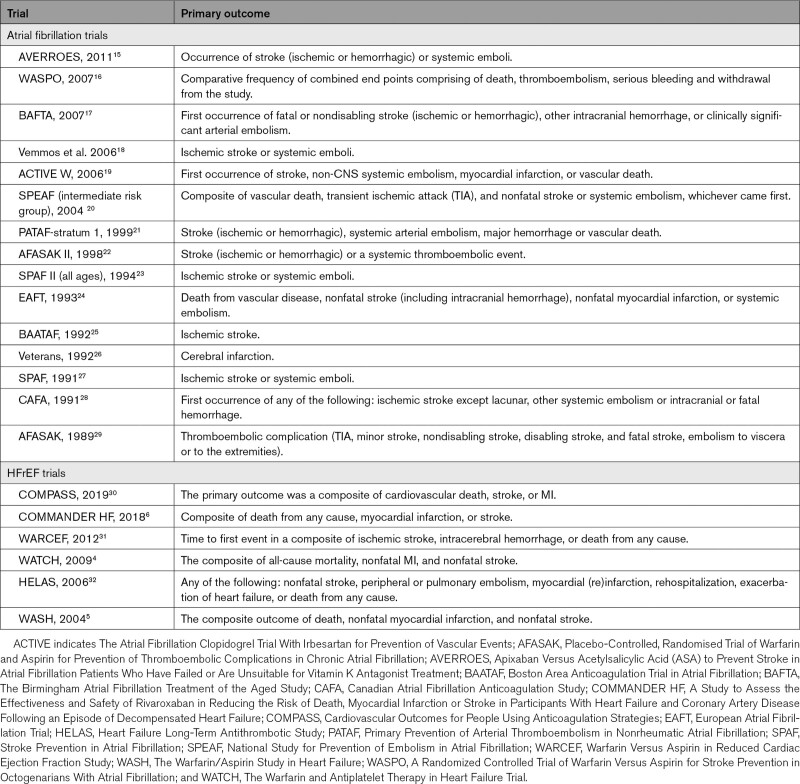
Primary Outcome Measures

### Data Synthesis and Analysis

A descriptive analysis of trial methodology and definitions of primary outcomes are reported in the Table and Table II in the Data Supplement.

Baseline characteristics are reported in Table III in the Data Supplement. We calculated the odds ratio (OR) and 95% CIs for each outcome of interest from individual studies. Weighted pooled treatment effects were calculated overall and individually for atrial fibrillation and HFrEF trials using restricted maximum likelihood estimation to fit a random effects meta-analysis model. Restricted maximum likelihood estimation was chosen because it has been shown to be less biased than the DerSimonian-Laird estimator.^33,34^ For outcomes with trials which had zero events (eg, hemorrhagic stroke and fatal hemorrhage), a one over reciprocal continuity correction sensitivity analysis was performed. Our objective was to determine the difference in treatment effect of oral anticoagulation between trials in atrial fibrillation and trials in HFrEF populations. We statistically tested for difference in treatment effect by (1) estimating the I^2^ statistic among all trials (ie, both populations), a measure of variability across studies due to heterogeneity and (2) testing a *P* for interaction between atrial fibrillation trials and HFrEF trials. We adopted a conservative approach to setting a threshold for a difference in treatment effect, namely (1) if I^2^ of >40% for all trials, and heterogeneity was explained by trial population (ie, change in I^2^ with separate meta-analytic estimates) and/or (2) evidence of statistical heterogeneity by trial population with a *P*-interaction <0.1.^35^ Publication bias was assessed using a funnel plot (Figures I and II in the Data Supplement). Summary estimates were calculated for atrial fibrillation trials, HFrEF trials, and all trials combined. Statistical analysis was performed using the Metafor package on R Statistical Software (Version 3.6.2).^34^ A priori subgroup sensitivity analyses were performed for trials deemed at low risk of bias, trials where aspirin was the comparator, trials targeting an INR range between 2 and 3.5. Post hoc sensitivity analyses were performed on the derived composite outcomes of (1) a major adverse cardiovascular event (MACE) outcome and (2) nonfatal major cardiovascular events.

### Risk of Bias Assessment

We used the Cochrane risk-of-bias tool for randomized trials (RoB 2) to assess methodological quality of eligible trials.^36^ Trials were assessed on 5 domains: randomization process, deviations from intended interventions, missing outcome data, measurement of the outcome and selection of the reported result. Risk of bias assessments was performed independently by reviewers (C.R. and E.L.) and disagreements were resolved by a third reviewer (R.M.). Studies were deemed at high risk of bias overall if one or more domains were rated as high, or if multiple domains were judged to have some concerns in a way that substantially lower confidence in the result.^36^ Risk of bias summary tables were created (Figures III through IV and Table IV in the Data Supplement).

## Results

The systematic search of articles published before November 2019, identified 2162 records. Following title and abstract screening, 68 were considered potentially relevant. After application of eligibility criteria to full text review, 21 trials (n=29 198) were included, of which 15 (n=19 332) were in atrial fibrillation and 6 (n=9866) were in HFrEF (PRISMA flow, Figure V in the Data Supplement and PRISMA checklist, Table V in the Data Supplement). All trials reported all stroke,^4–6,15–32^ 18 studies reported ischemic stroke,^4–6,15–28,31,37^ and 16 reported hemorrhagic stroke on follow-up.^4–6,15,17–22,24–27,31,37^

### Study Characteristics

The mean duration of follow-up was 23 months (23.1 months for atrial fibrillation trials, 23.9 months for HFrEF trials). The intervention group was warfarin or coumarin derivative for seventeen studies,^4,5,16–19,21–29,31,32^ acenocoumarol in 1 study,^20^ low-dose rivaroxaban for 2 studies,^6,30^ and apixaban for 1 study.^15^

Of included trials, 7 were double-blind (3 atrial fibrillation trials,^15,26,28^ 4 HFrEF trials^5,6,30,32^), 5 were blinded for the control group only with an open label design for anticoagulant group (3 atrial fibrillation trials,^24,27,29^ 2 HFrEF trials^4,5^), the remaining 9 atrial fibrillation trials were open label.^16–23,25^ Nineteen trials used a composite primary outcome (96.7% of total population).^4–6,15–24,27–32^ The components of the composite end points varied between atrial fibrillation trials and HFrEF trials, with 5 HFrEF trials (95.1% of HFrEF population) including all-cause mortality in their primary composite end point, compared with 1 atrial fibrillation trial (0.38% of atrial fibrillation population). Eight atrial fibrillation trials (48.8% of atrial fibrillation population) employed a primary outcome of stroke with/without systemic arterial thromboembolism, versus none of the HFrEF trials (Table).

### Risk of Bias

Risk of bias was assessed for 21 trials (Figures III and IV and Table IV in the Data Supplement). Risk of bias was deemed to be low in fifteen trials, some concerns in 3 trials, and high risk in 3 trials. The randomization process lead to some concerns for 3 atrial fibrillation studies.^18,25,28^ Measurement of outcome measures were deemed to be ‘high risk’ of bias for 1 atrial fibrillation trial^29^ and ‘some concerns’ for 1 atrial fibrillation trial.^18^ Publication bias was assessed using contour enhanced funnel plots, which were symmetrical around the point estimate for both atrial fibrillation trials and HFrEF trials (Figures I and II in the Data Supplement).

### Oral Anticoagulation and All Stroke

**Figure 1.**
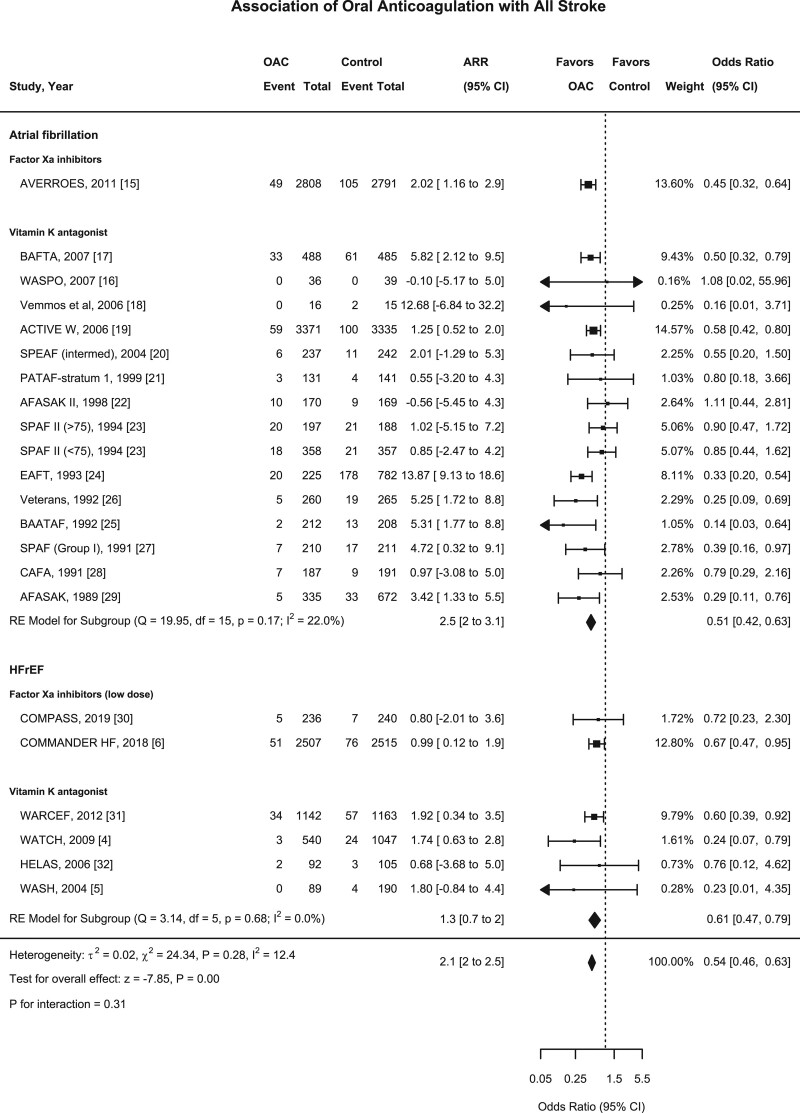
**Forest plot demonstrating the association of oral anticoagulant and all stroke.** The squares and bars represent the mean values and 95% CIs of the effect sizes, while the area of the squares reflects the weight of the studies. The combined effects appear as diamonds and the vertical dashed line represents the line of no effect. ARR indicates absolute risk reduction; HFrEF, heart failure with reduced ejection fraction; and OAC, oral anticoagulant.

**Figure 2.**
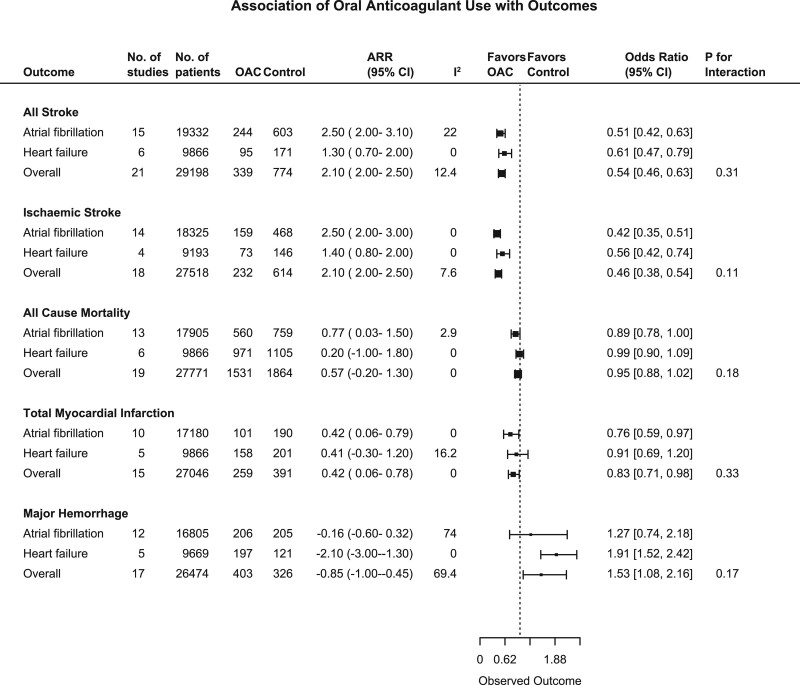
**Combined forest plot showing outcomes: all stroke, ischemic stroke, all-cause mortality, total myocardial infarction, and major hemorrhage.** The analysis is divided by population group; overall, atrial fibrillation trials, heart failure with reduced ejection fraction trials. The squares and bars represent the mean values and 95% CIs of the effect sizes, while the area of the squares reflects the weight of the studies. ARR indicates absolute risk reduction; and OAC, oral anticoagulant.

**Figure 3.**
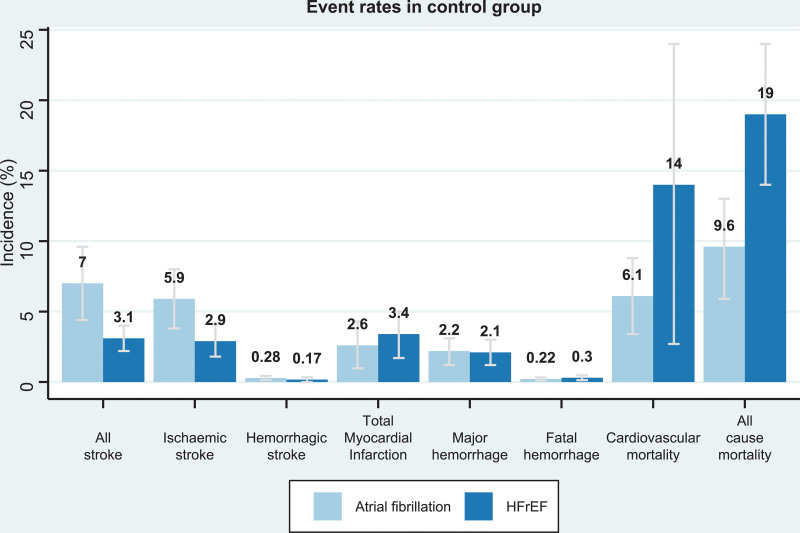
**Bar chart depicting the incidence rates of outcomes within the control group.** The light blue column represents atrial fibrillation trials, the dark blue represents heart failure with reduced ejection fraction (HFrEF) trials. The *y* axis represents percentage of trial population.

Among all 21 trials (n=29 198), there were 1113 stroke events during follow-up, 339 events in the oral anticoagulant group and 774 events in the control group.^4–6,15–32^ Oral anticoagulation compared with control was associated with a significant reduction in all stroke (2.8% versus 5.8% over a mean trial follow-up of 1.92 years; OR, 0.54 [95% CI, 0.46–0.63]; Absolute Risk Reduction [ARR], 2.1% [2–2.5]; Figures 1 and 2), with a consistent effect among trials (I^2^=12.4%). The association of oral anticoagulation and all stroke was similar for atrial fibrillation trials (OR, 0.51 [95% CI, 0.42–0.63]; ARR, 2.5% [2–3.1]) and HFrEF trials (OR, 0.61 [95% CI, 0.47–0.79]; ARR, 1.3% [0.7–2]; *P* interaction=0.31; Figures 1 and 2). The baseline incidence of all stroke in the control group was 7% in atrial fibrillation trials compared with 3.1% in HFrEF trials (Figure 3).

### Oral Anticoagulation and Ischemic Stroke

**Figure 4.**
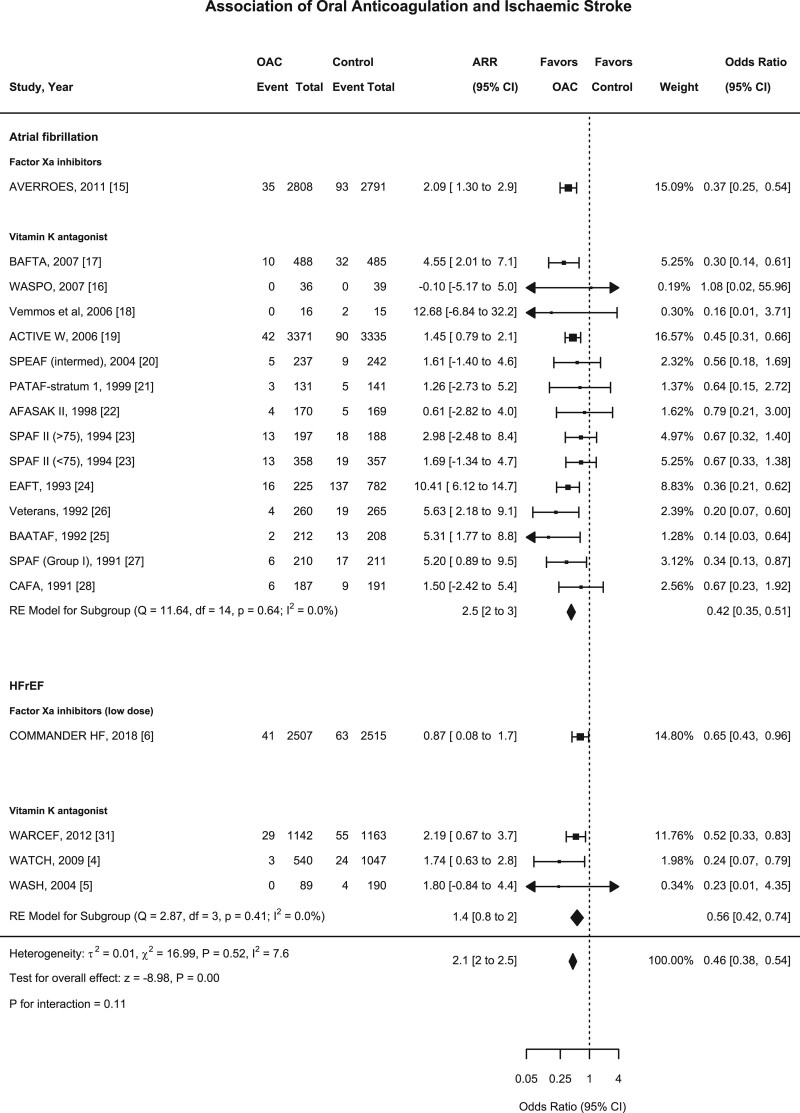
**Forest plot demonstrating the association of oral anticoagulant and ischemic stroke.** The squares and bars represent the mean values and 95% CIs of the effect sizes, while the area of the squares reflects the weight of the studies. The combined effects appear as diamonds and the vertical dashed line represents the line of no effect. ARR indicates absolute risk reduction; and HFrEF, heart failure with reduced ejection fraction; and OAC, oral anticoagulant.

Among eighteen studies (n=27 518), there were 846 ischemic stroke events during follow-up, 232 events in the oral anticoagulant group and 614 events in the control group.^4,5,15–28,31,37^ Oral anticoagulation compared with control was associated with a significant reduction in ischemic stroke (1.9% versus 5.2% over a mean of 1.93 years; OR, 0.46 [95% CI, 0.38–0.54]; ARR, 2.1% [2–2.5]; Figures 2 and 3), with a consistent effect among trials (I^2^=7.6%). The association of oral anticoagulation and ischemic stroke was similar for atrial fibrillation trials (OR, 0.42 [95% CI, 0.35–0.51]; ARR, 2.5% [2–3]) and the HFrEF trials (OR, 0.56 [95% CI, 0.42–0.74]; ARR, 1.4% [0.8–2]; *P* interaction=0.11; Figures 2 and 4). The baseline incidence of ischemic stroke in the control group was 5.9% in atrial fibrillation trials compared with 2.9% in HFrEF trials (Figure 4).

### Oral Anticoagulation and Hemorrhagic Stroke

Among 16 studies (n=26 040), there were 79 hemorrhagic stroke events during follow-up, including 43 events in the oral anticoagulant group and 36 events in the control group.^4–6,15–22,24–27,31,37^ Oral anticoagulation compared with control was not associated with a significant increase in hemorrhagic stroke (0.29% versus 0.19% over a mean of 1.96 years; OR, 1.23 [95% CI, 0.76–1.99]; absolute risk increase [ARI], 0.068%, 0.067 to −0.2; Figure VI in the Data Supplement), with a consistent effect among trials (I^2^=6.0%). The association of oral anticoagulation and hemorrhagic stroke was similar for atrial fibrillation trials (OR, 1.24 [95% CI, 0.69 to 2.25]; ARI, 0.092% [−0.086 to 0.3]) and HFrEF trials (OR, 1.20 [95% CI, 0.47 to 3.07]; ARI, 0.024% [0.2 to −0.18]; *P* interaction=0.95; Figure VI in the Data Supplement).

### Oral Anticoagulation and Mortality

Among 19 studies (n=27 771), there were 3395 deaths during follow-up including 1531 participants in the oral anticoagulant group and 1864 participants in the control group.^4,6,15–24,26–28,30–32^ Oral anticoagulation compared with control was not significantly associated with a reduction in all-cause mortality (11% versus 12% over a mean of 1.91 years; OR, 0.95 [95% CI, 0.88 to 1.02]; ARR, 0.57% [−0.2 to 1.3]), with a consistent effect among trials (I^2^=0%) (Figure 3, Figure VII in the Data Supplement). There was no evidence of a statistically significant difference (I^2^=0, *P* interaction=0.18), the association of oral anticoagulation and all-cause mortality was similar for atrial fibrillation trials (OR, 0.89 [95% CI, 0.78–1.00]; ARR, 0.77% [0.03–1.5]) and HFrEF trials (OR, 0.99 [95% CI, 0.90 to 1.09]; ARR 0.2%, −1 to 1.8; Figure 2, Figure VII in the Data Supplement). Oral anticoagulation compared with control was associated with a borderline significant reduction in cardiovascular mortality; OR, 0.90 [95% CI, 0.81–0.99] (Figure VIII in the Data Supplement). The incidence of all-cause mortality in the control group was 9.6% in atrial fibrillation trials compared with 19% in HFrEF trials (Figure 3).

### Oral Anticoagulation and Myocardial Infarction

Sixteen studies (n=27 046) reported 650 myocardial infarction events during follow-up.^4–6,15,17–20,22–24,26,27,30–32^ Oral anticoagulation compared with control was significantly associated with a reduction in myocardial infarction (2.2% versus 2.9% over a mean of 1.96 years; OR, 0.83 [95% CI, 0.71–0.98]; ARR, 0.42% [0.06–0.78]; Figure 2, Figure IX in the Data Supplement). There was no evidence of a statistically significant difference (I^2^=0%, *P* interaction=0.33) between the association of oral anticoagulation and myocardial infarction, although there was a significant reduction in atrial fibrillation populations (OR, 0.76 [95% CI, 0.59 to 0.97]; ARR, 0.42% [0.06 to 0.79]) but not HFrEF trials (OR, 0.91 [95% CI, 0.69 to 1.20]; ARR, 0.41% [ −0.3 to 1.2]; Figure 2, Figure IX in the Data Supplement).

### Oral Anticoagulation and Bleeding Outcomes

#### Major and Fatal Hemorrhage

Among 17 studies (n=26 474), there were 729 major hemorrhage events during follow-up.^4–6,15–22,24,26–28,30,31^ Oral anticoagulation compared with control was significantly associated with an increase in major hemorrhage (3.4% versus 2.1% over a mean of 1.85 years; OR, 1.53 [95% CI, 1.08–2.16]; ARI, 0.85% [0.45–1]), with an inconsistent effect among trials (I^2^=69.4%; Figure 2, Figure X in the Data Supplement). There was no evidence of a statistically significant difference between the association of oral anticoagulation and major hemorrhage for atrial fibrillation (OR, 1.27 [95% CI, 0.74 to 2.18]; ARI, 0.16% [−0.32 to 0.6]) and HFrEF trials (OR, 1.91 [95% CI, 1.52 to 2.42]; ARI, 2.1% [1.3 to 3]; *P*-interaction=0.17; Figure 2, Figure X in the Data Supplement), or fatal hemorrhage (Figure XI in the Data Supplement).

### Oral Anticoagulation and Original Primary Outcomes Reported in Trials

Among 21 studies (n=29 198), there were 3823 events during follow-up including 1570 events in the oral anticoagulation group and 2253 events in the control group. For the original primary outcome, 19 trials reported a composite outcome^4–6,15–24,27–32^ and 2 reported stroke as a single primary outcome.^25,26^ Definitions of composite outcomes varied between trials (Table I in the Data Supplement). The association of oral anticoagulation and original primary outcome differed for atrial fibrillation trials (OR, 0.58 [95% CI, 0.47 to 0.72]; ARR, 3.1% [2 to 3.8]) and HFrEF trials (OR, 0.93 [95% CI, 0.85 to 1.02]; ARR, 1.3% [−0.4 to 3]; *P*-interaction=0.0001; Figure XII in the Data Supplement).

### Sensitivity Analyses

Sensitivity analysis including only trials at low risk of bias, aspirin as control, warfarin as intervention, and a targeted INR target of 2 to 3.5 did not materially alter the findings for all stroke, ischemic stroke or hemorrhagic stroke and mortality (Figure XIII through XVI in the Data Supplement).

### Post Hoc Standardized Composite Outcome Measure

#### MACE Composite Outcome

Among 13 studies (n=25 075), in which we derived major adverse cardiovascular events (MACE), oral anticoagulation compared with control was associated with a significant reduction in MACE composite outcome (OR, 0.86 [95% CI, 0.740–0.996]; ARR, 1.4% [0.6–2.3]), with statistical evidence of heterogeneity among all trials (I^2^=65.1%, I^2^ for atrial fibrillation trials=73.8%, I^2^ for HFrEF trials=0%). The association of oral anticoagulation and MACE composite was not significant for atrial fibrillation trials (OR, 0.82 [95% CI, 0.63 to 1.07]; ARR, 1.4% [0.5 to 2.3]) and HFrEF trials (OR, 0.92 [95% CI, 0.84 to 1.01]; ARR, 1.4% [−0.2 to 3.1]; *P*-interaction=0.43; Figure XVII in the Data Supplement).

In HFrEF studies (n=5),^4–6,31,32^ oral anticoagulation compared with control was associated with a significant reduction in nonfatal cardiovascular events (OR, 0.75 [95% CI, 0.62–0.89], I^2^=0%; Figure XVIII in the Data Supplement).

## Discussion

This systematic review and meta-analysis, which included 21 trials with 29 198 participants for the primary outcome analysis, found that the association of oral anticoagulation with stroke risk is consistent for patients with atrial fibrillation and HFrEF in sinus rhythm. We found no evidence of statistically significant differences in the association of oral anticoagulation with ischemic stroke, hemorrhagic stroke, myocardial infarction or fatal bleeding between populations. There were differences in incidence of clinical events between atrial fibrillation and HFrEF populations, with markedly higher mortality in HFrEF trials.

This updated meta-analysis extends findings from previous meta-analyses that have examined both populations separately, by including a larger number of randomized clinical trials including a recently published study^30^ and providing comparative estimates of treatment effects in atrial fibrillation and HFrEF populations within a combined meta-analysis. While previous meta-analyses specific to trials of atrial fibrillation or HFrEF have reported similar estimates to the current analysis,^38–41^ this meta-analysis considered all trials together, which allowed us to determine the homogeneity of treatment effects across both populations. We think that our meta-analysis provides an innovative perspective through including clinical trials from both populations in a single analysis, lending insights that may not be apparent through indirect comparisons of individual meta-analyses. Moreover, it permitted us to explore the effect of methodological differences (eg, outcome measures) between trials of patients with atrial fibrillation and those with HFrEF. We observed a major difference in the primary outcome measures employed in trials of patients with atrial fibrillation, versus HFrEF, which have likely contributed to differing conclusions of the relative efficacy of oral anticoagulation. A key difference is that atrial fibrillation trials more often prioritized stroke in primary outcome measures (Table). We observed an identical magnitude of association of oral anticoagulation and stroke risk in trials of atrial fibrillation and HFrEF, which permits speculation that current guideline recommendations may be different if trials of HFrEF employed the same primary outcome measure as atrial fibrillation trials.

All HFrEF trials included mortality in a primary composite outcome measure, compared with only 1 atrial fibrillation trial.^16^ Although we did not identify a statistically significant difference in the association between oral anticoagulation and mortality or cardiovascular mortality (Figure 3, Figures VII and VIII in the Data Supplement), we report a reduced risk of cardiovascular mortality in atrial fibrillation, but no significant association in HFrEF trials. Our findings suggest, but do not confirm, that there may be differing contributions of thromboembolic causes of death between these 2 populations. Moreover, the mortality rate in the HFrEF trials was considerably higher than in the atrial fibrillation trials, which further diluted the treatment effect on nonfatal cardiovascular events. Use of composite outcome measures in randomized clinical trials has been challenged in recent years, with the emergence of criteria for valid composite outcomes based on consistency of patient-reported importance and expected consistency in frequency of treatment effect across individual components of composite outcomes.^42^ While all-cause mortality is of major clinical importance, it appears to be unaffected by oral anticoagulation in patients with HFrEF and would not satisfy this criterion for inclusion in a valid composite outcome.^43,44^

Antithrombotic cardiovascular guidelines offer diverse recommendations in HFrEF, ranging from weak recommendations in HFrEF with moderate to high stroke risk,^45^ to weak recommendations against oral anticoagulant therapy^8,46^ (Table VI in the Data Supplement). The case for prescribing oral anticoagulation in patients with HFrEF rests on the significant reduction in stroke incidence, and clinical decision-making for individual patients will depend on the absolute risk of stroke. In contrast to atrial fibrillation (eg, CHA_2_DS_2_-VASc score), there is no widely used score to quantify the risk of stroke in patients with HFrEF, but use of oral anticoagulation in obviously high-risk populations (eg, prior cardioembolism) would be supported by our findings, provided the competing risk of major bleeding is acceptable. Further evidence evaluating oral anticoagulation in RCTs in HFrEF population is warranted, particularly for primary prevention where there is more uncertainty regarding risk-benefit. Based on our findings, use of oral anticoagulation would seem reasonable among patient populations who are considered at high risk of cardioembolic stroke, such as those with recent ischemic stroke or transient ischemic attack, where HFrEF is implicated as causal. Although the risk of bleeding in patients with HFrEF is a commonly cited reason to avoid oral anticoagulation,^40^ our findings did not reveal major differences in the risk of major or fatal bleeding in patients with atrial fibrillation compared with those with HFrEF (*P* for interaction=0.17). Although we note that oral anticoagulation was associated with significant increase in major hemorrhage in the HFrEF population. Anticoagulation was not associated with a significant increase in fatal hemorrhage in either population.

For many stroke physicians, anticoagulation decision-making for patients with atrial fibrillation is clear, while the decision in patients with heart failure is more challenging because of inconsistent guideline recommendations.^7,8^ Our analysis offers relevant context for the benefit of oral anticoagulation in HFrEF by comparing directly to a population where the benefit is universally accepted and consistently recommended in guidelines.^1^ Clinical context (eg, prior ischemic stroke), and patient preference, should determine selective prescribing of low-dose or treatment-dose regimens of oral anticoagulant therapy in patients with HFrEF.

### Limitations of Our Study

This study has several limitations. First, intervention groups consisted of a combination of 2 different types of oral anticoagulant therapy, vitamin K antagonists, and factor Xa inhibitors, and at differing intensities including low-dose rivaroxaban in the COMPASS (Cardiovascular Outcomes for People Using Anticoagulation Strategies) and COMMANDER HF trials (A Study to Assess the Effectiveness and Safety of Rivaroxaban in Reducing the Risk of Death, Myocardial Infarction or Stroke in Participants With Heart Failure and Coronary Artery Disease Following an Episode of Decompensated Heart Failure).^6,30^ We note that there are a smaller number of trials evaluating DOACs than vitamin K antagonists in HFrEF, which may warrant further investigation. Second, the INR targets varied between studies, although a sensitivity analysis restricting INR target (2–3.5) demonstrated similar results. Third, definitions of both original primary outcomes of individual trials and major hemorrhage varied among studies. This limitation is expected to be most problematic when comparing absolute rates of bleeding among trials. We have included the definition of major hemorrhage adopted by each trial in a Table in the Data Supplement for clarity. Fourth, there were low event rates for some outcomes (eg, hemorrhagic stroke), with consequence imprecise summary estimates. Although meta-analysis of rare events should be interpreted with caution, we completed a one over reciprocal continuity correction sensitivity analysis, which did not alter our findings results.^47^ Fifth, the number of patients included in atrial fibrillation trials were higher than in HFrEF trials, which limits the validity of some comparisons.^4,5,31^ Sixth, our primary outcome and research hypothesis related to all stroke, all other statistically significant results should be considered as secondary outcomes subject to the limitations of multiple testing.

## Conclusions

In this meta-analysis of randomized clinical trials, oral anticoagulation compared with control was significantly associated with a lower incidence of stroke in patients with atrial fibrillation or HFrEF. Differences in the primary outcomes employed by trials in HFrEF, compared with atrial fibrillation, may have contributed to differing conclusions of the relative efficacy of oral anticoagulation, particularly the inclusion of all-cause mortality in most HFrEF trials.

## Acknowledgments

C. Reddin and E. Loughlin were responsible for data collection. C. Reddin and C. Judge performed the analysis. All authors contributed to data interpretation and critical revision of the report.

## Sources of Funding

C. Judge was supported by the Irish Clinical Academic Training (ICAT) Programme, the Wellcome Trust and the Health Research Board (grant number 203930/B/16/Z), the Health Service Executive, National Doctors Training and Planning, and the Health and Social Care, Research and Development Division, Northern Ireland. Dr O’Donnell was supported by the European Research Council (COSIP grant, 640580). The funding source had no role in the design and conduct of the study or the publication decision.

## Disclosures

None.

## Supplemental Materials

Online Methods

Online Tables I–VI

Online Figures I–XVIII

## Supplementary Material


