# Association of oral anticoagulation with stroke in atrial fibrillation or heart failure: a comparative meta-analysis

**DOI:** 10.1161/STROKEAHA.120.033910

**Published:** 2021-07-20

**Authors:** Catriona Reddin, Conor Judge, Elaine Loughlin, Robert Murphy, Maria Costello, Alberto Alvarez, John Ferguson, Andrew Smyth, Michelle Canavan, Martin J. O’Donnell

**Affiliations:** 1 HRB-Clinical Research Facility, National University of Ireland Galway, Galway, Ireland; 2 Galway University Hospital, Newcastle Road, Galway, Ireland; 3 Translational Medical Device Laboratory, National University of Ireland Galway, Galway, Ireland; 4 Wellcome Trust – HRB, Irish Clinical Academic Training

## Abstract

**Background and Purpose:**

Atrial fibrillation and heart failure with reduced ejection fraction(HFrEF) are common sources of cardioembolism. While oral anticoagulation is strongly recommended for atrial fibrillation, there are marked variations in guideline recommendations for HFrEF due to uncertainty about net clinical benefit.

This systematic review and meta-analysis evaluates the comparative association of oral anticoagulation with stroke and other cardiovascular risk in populations with atrial fibrillation or HFrEF in sinus rhythm, and identify factors mediating different estimates of net clinical benefit.

**Methods:**

PubMed and Embase were searched from database inception to November 20^th^, 2019 for randomized clinical trials comparing oral anticoagulation to control. A random-effects meta-analysis was used to estimate a pooled treatment-effect overall, and within atrial fibrillation and HFrEF trials. Differences in treatment effect were assessed by estimating I^2^ among all trials and testing the between-trial-population P-interaction. The primary outcome measure was all stroke. Secondary outcome measures were ischaemic stroke, hemorrhagic stroke, mortality, myocardial infarction, and major haemorrhage.

**Results:**

Twenty-one trials were eligible for inclusion, 15(n=19,332) in atrial fibrillation(mean follow-up:23.1 months) and 6(n=9,866) in HFrEF(mean follow-up:23.9 months). There were differences in primary outcomes between trial populations, with all-cause mortality included for 95.2% of HFrEF trial population versus 0.38% for atrial fibrillation. Mortality was higher in controls groups of HFrEF populations(19.0% versus 9.6%) but rates of stroke lower(3.1% versus 7.0%) compared to atrial fibrillation. The association of oral anticoagulation with all stroke was consistent for atrial fibrillation(OR 0.51;95%CI,0.42-0.63) and HFrEF(OR 0.61;95%CI,0.47-0.79)(I^2^=12.4%; P-interaction=0.31). There were no statistically significant differences in the association of oral anticoagulation with cardiovascular events, mortality or bleeding between populations.

**Conclusions:**

The relative association of oral anticoagulation with stroke risk, and other cardiovascular outcomes, is similar for patients with atrial fibrillation and HFrEF. Differences in the primary outcomes employed by trials in HFrEF, compared to atrial fibrillation, may have contributed to differing conclusions of the relative efficacy of oral anticoagulation.

## Introduction

Atrial fibrillation and heart failure with reduced ejection fraction (HFrEF) are common causes of cardioembolic stroke. Oral anticoagulation is strongly recommended (Grade 1A) for patients with atrial fibrillation to reduce the risk of ischaemic stroke ^1–3^. In contrast, clinical trials in patients with HFrEF in sinus rhythm have not reported superiority of oral anticoagulation versus antiplatelet therapy or control for cardiovascular prevention ^4–6^, and guideline recommendations are inconsistent ^7,8^. For example, the Heart Failure Society of America recommends anticoagulation with warfarin, with an International Normalised Ratio (INR) target of 2-3 for patients with HFrEF and a history of thromboembolism ^7,9^. Conversely, the American Heart Association make a level B recommendation against anticoagulation in heart failure in the absence of atrial fibrillation ^8^. Several factors may explain the apparent difference in the efficacy, or interpretation of efficacy from clinical trials, of oral anticoagulation in atrial fibrillation compared to HFrEF in sinus rhythm. First, the mechanism of ischaemic stroke may differ between populations, and patients with HFrEF may have a higher prevalence of competing stroke aetiologies, for which oral anticoagulation is not superior to antiplatelet therapy (e.g. small vessel disease). Second, the risks associated with oral anticoagulant therapy may differ in patients with HFrEF than in patients with atrial fibrillation, for example the risk of intracerebral hemorrhage, which may offset benefits in the reduction of ischaemic stroke ^10,11^. Third, differences in the primary outcome measures between trials of patients with HFrEF (which mostly used composite outcomes, including all-cause mortality) and trials of patients with atrial fibrillation (which mostly used all stroke) may account for the observed difference in efficacy of oral anticoagulation. In addition, there may be differences in the relative contribution of stroke to a composite outcome between populations.

While prior meta-analyses have examined the effectiveness of oral anticoagulation in patients with atrial fibrillation or HFrEF individually, our meta-analysis specifically evaluates the comparative association of oral anticoagulation with incidence of stroke, other cardiovascular events and mortality in populations with atrial fibrillation compared to populations with HFrEF in sinus rhythm.

## Methods

We performed a systematic review and meta-analysis, adhering to the Cochrane Collaboration Guidelines and reported our findings according to the Preferred Reporting Items for Systematic Reviews and Meta-Analyses (PRISMA) Guidelines ^12,13^. The meta-analysis was registered with the International Prospective Register of Systematic Reviews (PROSPERO identifier: CRD42020153013). The data that support the findings of this study are available from the corresponding author upon reasonable request.

### Data Sources and Search Strategy

We systematically searched PubMed and Embase databases from database inception to November 20^th^, 2019. The search terms included are detailed in the Supplementary Appendix (eMethods I). The search strategy was peer-reviewed by an information specialist. Following removal of duplicates, titles and abstracts were screened by two reviewers (CR and EL) using the Rayann web application ^14^. The reference lists of included trials and other published meta-analyses were also reviewed. Full texts of remaining articles were independently assessed by two reviewers (CR and EL), with eligibility based on predetermined criteria. Disagreements were resolved by consensus, where a resolution was not reached by discussion, a consensus was reached through a third reviewer (CJ).

### Eligibility Criteria

Studies were considered eligible if they: [1] were randomized clinical trials; [2] included adults greater than 18 years; [3] evaluated oral anticoagulation compared with control; and [4] reported stroke events (at least one of the following: all stroke, ischaemic stroke or hemorrhagic stroke). Control was defined as antiplatelet, placebo or no antithrombotic treatment.

### Data Extraction/Measurements

Data were extracted independently by two authors (CR and EL) using a standardized pre-determined data collection form. For each study, we extracted the title, year of publication, oral anticoagulant (including dose/target), antiplatelet (including dose, where applicable), active and control numbers, all stroke, ischaemic stroke, hemorrhagic stroke, all-cause mortality, cardiovascular mortality, total myocardial infarction, major hemorrhage, fatal hemorrhage, and original primary outcomes of individual trials. We did not pre-specify a definition for stroke or major hemorrhage. Data were compared for inconsistencies and merged into a pre-final dataset which was checked independently by two other reviewers (CJ and MC).

### Outcomes

The primary outcome measure was all stroke. The secondary outcome measures were ischaemic stroke, hemorrhagic stroke, all-cause mortality, cardiovascular mortality, total myocardial infarction, major hemorrhage, fatal hemorrhage, and original primary outcomes of individual trials. The definition of original primary outcomes of individual trials and major hemorrhage differed among trials (Table 1, eTable I).

### Data Synthesis and Analysis

A descriptive analysis of trial methodology and definitions of primary outcomes are reported in Table 1 and eTable II.

Baseline characteristics are reported in eTable III. We calculated the odds ratio (OR) and 95% confidence intervals (CI) for each outcome of interest from individual studies. Weighted pooled treatment effects were calculated overall and individually for atrial fibrillation and HFrEF trials using restricted maximum likelihood (REML) estimation to fit a random effects meta-analysis model. REML estimation was chosen because it has been shown to be less biased than the DerSimonian-Laird estimator ^33,34^. For outcomes with trials which had zero events (e.g. hemorrhagic stroke and fatal hemorrhage), a one over reciprocal continuity correction sensitivity analysis was performed. Our objective was to determine the difference in treatment effect of oral anticoagulation between trials in atrial fibrillation and trials in HFrEF populations. We statistically tested for difference in treatment effect by: [1] estimating the I^2^ statistic among all trials (i.e. both populations), a measure of variability across studies due to heterogeneity; and [2] testing a P for interaction between atrial fibrillation trials and HFrEF trials. We adopted a conservative approach to setting a threshold for a difference in treatment effect, namely an [1] if I^2^ of >40% for all trials, and heterogeneity was explained by trial population (i.e. change in I^2^ with separate meta-analytic estimates); and/or [2] evidence of statistical heterogeneity by trial population with a P-interaction <0.1 ^35^. Publication bias was assessed using a funnel plot (eFigure I-II). Summary estimates were calculated for atrial fibrillation trials, HFrEF trials, and all trials combined. Statistical analysis was performed using the Metafor package on R Statistical Software (Version 3.6.2) ^34^. A priori subgroup sensitivity analyses were performed for trials deemed at low risk of bias, trials where aspirin was the comparator, trials targeting an INR range between 2-3.5. Post-hoc sensitivity analyses were performed on the derived composite outcomes of: [1] a major adverse cardiac events (MACE) outcome, [2] non-fatal major cardiovascular events.

### Risk of Bias Assessment

We used the Cochrane risk-of-bias tool for randomized trials (RoB 2) to assess methodological quality of eligible trials ^36^. Trials were assessed on five domains: randomization process, deviations from intended interventions, missing outcome data, measurement of the outcome and selection of the reported result. Risk of bias assessments were performed independently by reviewers (CR and EL) and disagreements were resolved by a third reviewer (RM). Studies were deemed at high risk of bias overall if one or more domains were rated as high, or if multiple domains were judged to have “some concerns in a way that substantially lower confidence in the result” ^36^. Risk of bias summary tables were created (eFigure III-IV, eTable IV).

## Results

The systematic search of articles published before November 2019, identified 2,162 records. Following title and abstract screening, 68 were considered potentially relevant. After application of eligibility criteria to full text review, 21 trials (n=29,198) were included, of which 15 (n=19,332) were in atrial fibrillation and 6 (n=9,866) were in HFrEF (PRISMA flow eFigure V and PRISMA checklist, eTable V). All trials reported all stroke ^4–6,15–32^, 18 studies reported ischaemic stroke ^4–6,15–28,31,37^ and 16 reported hemorrhagic stroke on follow-up ^4–6,15,17–22,24–27,31,37^.

### Study Characteristics

The mean duration of follow-up was 23 months (23.1 months for atrial fibrillation trials, 23.9 months for HFrEF trials). The intervention group was warfarin or coumarin derivative for seventeen studies ^4,5,16–19,21–29,31,32^, acenocoumarol in one study ^20^, low-dose rivaroxaban for two studies ^6,30^ and apixaban for one study ^15^.

Of included trials, 7 were double-blind (3 atrial fibrillation trials ^15,26,28^, 4 HFrEF trials ^5,6,30,32^), 5 were blinded for the control group only with an open label design for anticoagulant group (3 atrial fibrillation trials ^24,27,29^, 2 HFrEF trials ^4,5^), the remaining 9 atrial fibrillation trials were open label ^16–23,25^. Nineteen trials used a composite primary outcome (96.7% of total population) ^4–6,15–24,27–32^. The components of the composite endpoints varied between atrial fibrillation trials and HFrEF trials, with 5 HFrEF trials (95.1% of HFrEF population) including all-cause mortality in their primary composite endpoint, compared to one atrial fibrillation trial (0.38% of atrial fibrillation population). Eight atrial fibrillation trials (48.8% of atrial fibrillation population) employed a primary outcome of stroke with/without systemic arterial thromboembolism, versus none of the HFrEF trials (Table 1).

### Risk of Bias

Risk of bias was assessed for twenty-one trials (eFigure III-IV, eTable IV). Risk of bias was deemed to be ‘low’ in fifteen trials, ‘some concerns’ in three trials, and ‘high risk’ in three trials. The randomization process lead to some concerns for three atrial fibrillation studies ^18,25,28^. Measurement of outcome measures were deemed to be ‘high risk’ of bias for one atrial fibrillation trial ^29^ and ‘some concerns’ for one atrial fibrillation trial ^18^. Publication bias was assessed using contour enhanced funnel plots, which were symmetrical around the point estimate for both atrial fibrillation trials and HFrEF trials (eFigure I,II).

### Oral Anticoagulation and All Stroke

**Figure 1:**
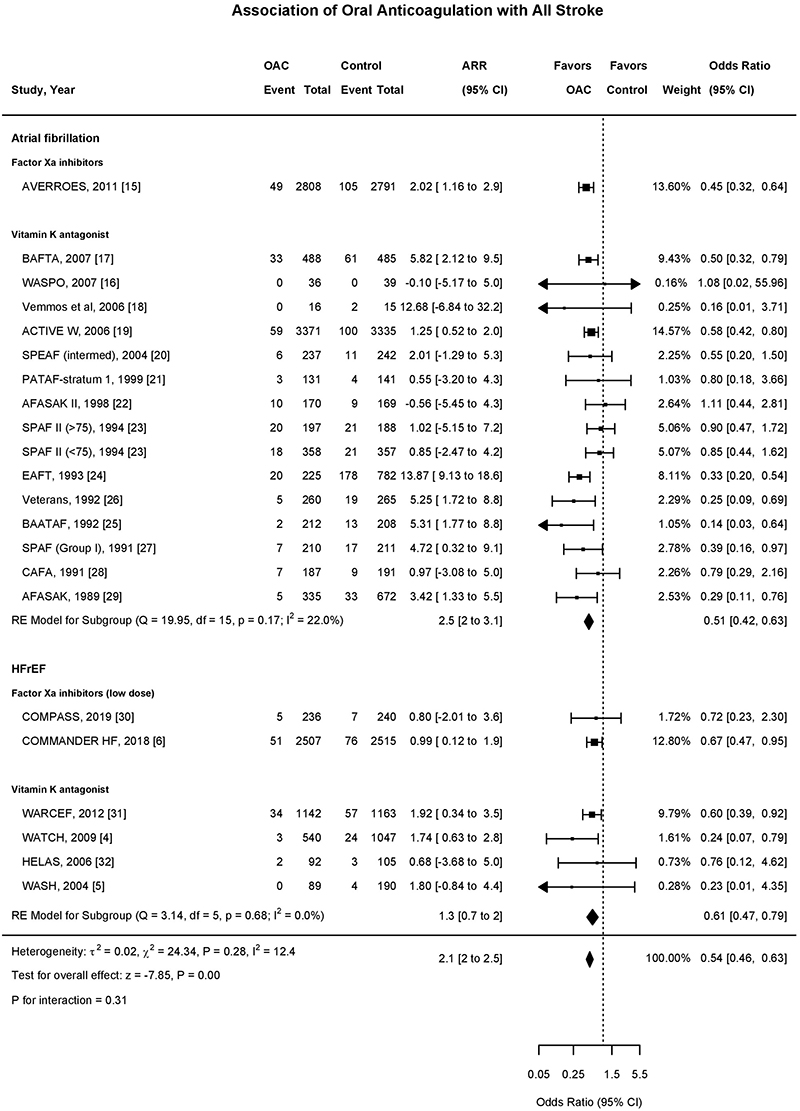
Association of Oral Anticoagulant Use with All Stroke Forest plot demonstrating the association of oral anticoagulant and all stroke. The squares and bars represent the mean values and 95% confidence intervals of the effect sizes, while the area of the squares reflects the weight of the studies. The combined effects appear as diamonds and the vertical dashed line represents the line of no effect. Int-Intervention, CI-Confidence Interval, ARR-Absolute risk reduction.

**Figure 2:**
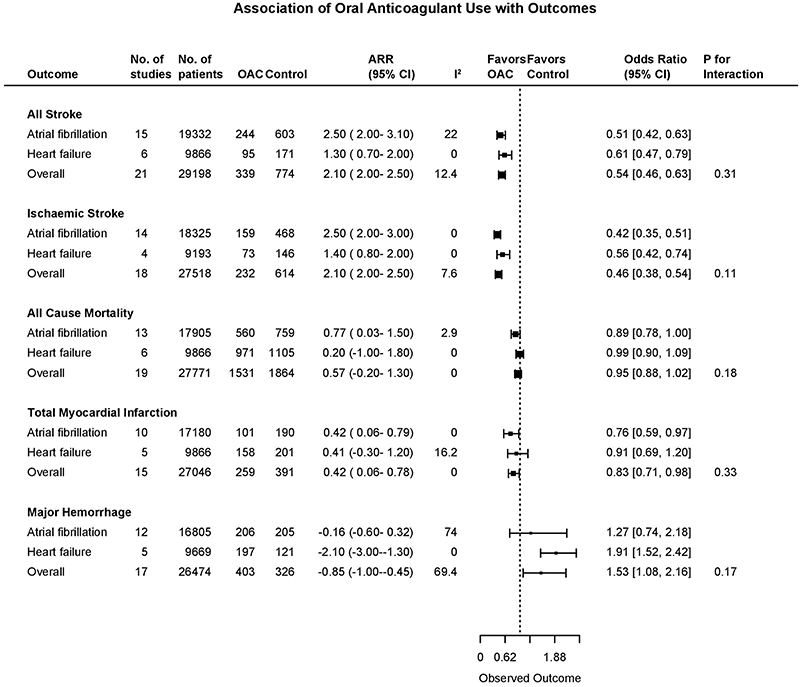
Association of Oral Anticoagulant Use with Outcomes Combined Forest Plot showing outcomes: all stroke, ischaemic stroke, all-cause mortality, total myocardial infarction, and major hemorrhage. The analysis is divided by population group; overall, atrial fibrillation trials, HFrEF trials. The squares and bars represent the mean values and 95% confidence intervals of the effect sizes, while the area of the squares reflects the weight of the studies. Int-Intervention, CI-Confidence Interval, ARR-Absolute risk reduction.

**Figure 3:**
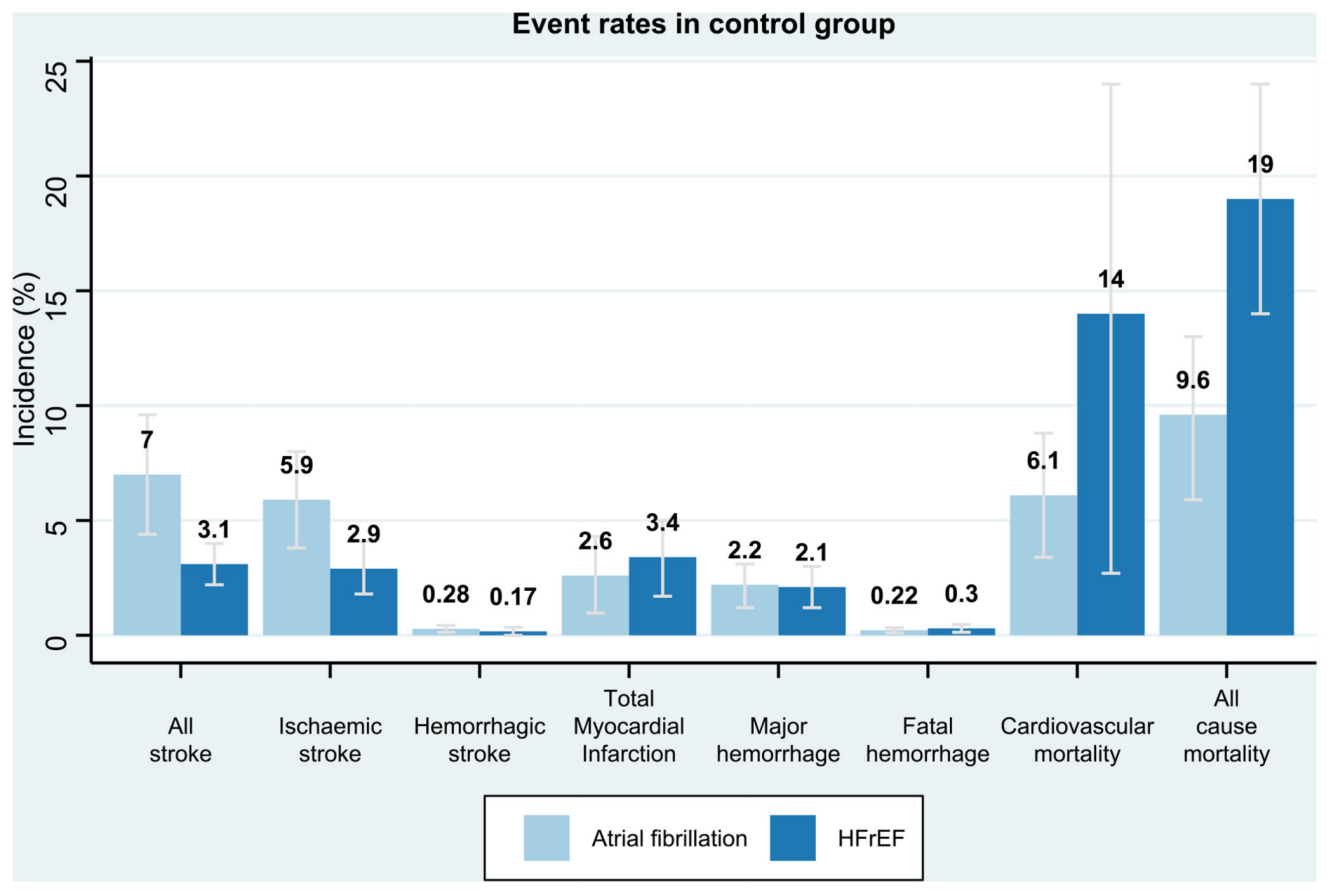
Event rates in control group Bar chart depicting the incidence rates of outcomes within the control group. The light blue column represents atrial fibrillation trials, the dark blue represents HFrEF trials. The y-axis represents percentage of trial population. HFrEF-Heart failure with reduced ejection fraction.

Among all twenty one trials (n=29,198), there were 1113 stroke events during follow-up, 339 events in the oral anticoagulant group and 774 events in the control group ^4–6,15–32^. Oral anticoagulation compared to control was associated with a significant reduction in all stroke (2.8% vs 5.8% over a mean trial follow-up of 1.92 years) (OR, 0.54;95%CI,0.46-0.63; Absolute Risk Reduction (ARR) 2.1%,2 to 2.5) (Figure 1,2), with a consistent effect among trials (I^2^=12.4%). The association of oral anticoagulation and all stroke was similar for atrial fibrillation trials (OR, 0.51;95%CI,0.42-0.63; ARR 2.5%,2 to 3.1) and HFrEF trials (OR, 0.61;95%CI,0.47-0.79; ARR 1.3%,0.7 to 2) (P-interaction=0.31) (Figure 1,2). The baseline incidence of all stroke in the control group was 7% in atrial fibrillation trials compared to 3.1% in HFrEF trials (Figure 3).

### Oral Anticoagulation and Ischaemic stroke

**Figure 4:**
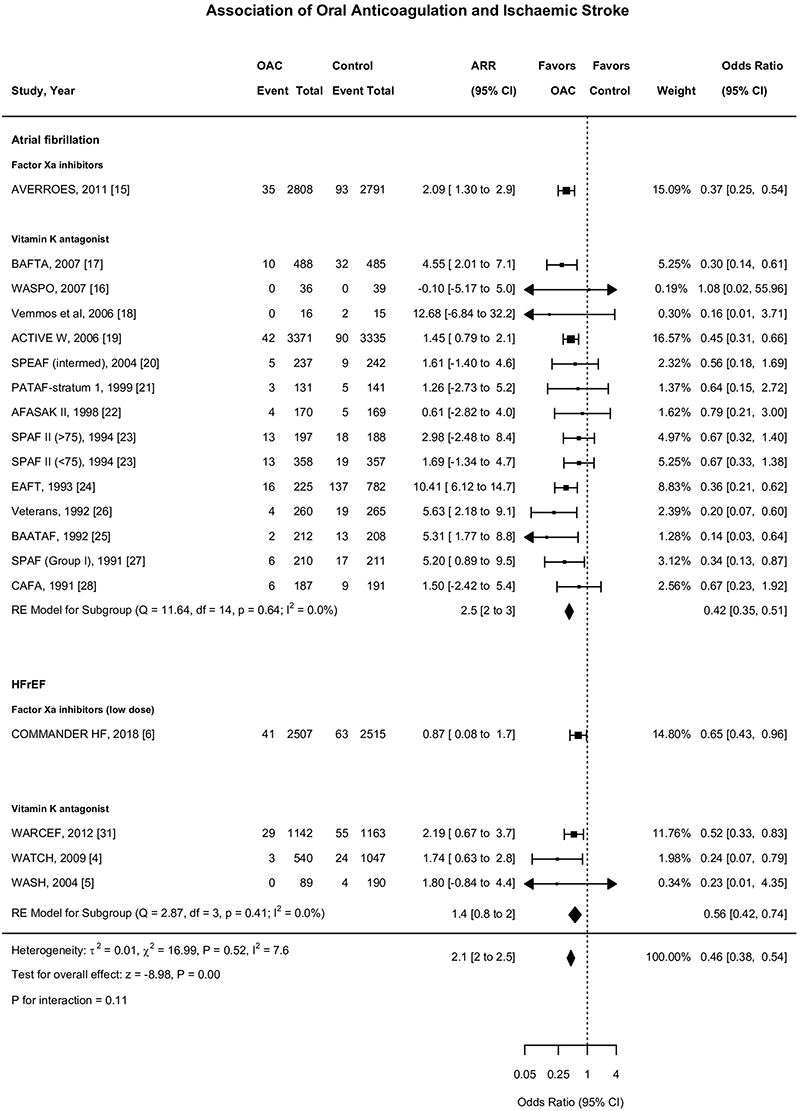
Association of Oral Anticoagulant Use with Ischaemic Stroke Forest plot demonstrating the association of oral anticoagulant and ischaemic stroke. The squares and bars represent the mean values and 95% confidence intervals of the effect sizes, while the area of the squares reflects the weight of the studies. The combined effects appear as diamonds and the vertical dashed line represents the line of no effect. Int-Intervention, CI-Confidence Interval, ARR-Absolute risk reduction.

Among eighteen studies (n=27,518), there were 846 ischaemic stroke events during follow-up, 232 events in the oral anticoagulant group and 614 events in the control group ^4,5,15–28,31,37^. Oral anticoagulation compared to control was associated with a significant reduction in ischaemic stroke (1.9% vs 5.2% over a mean of 1.93 years) (OR, 0.46;95%CI,0.38-0.54; ARR 2.1%,2 to 2.5) (Figure 2,3), with a consistent effect among trials (I^2^=7.6%). The association of oral anticoagulation and ischaemic stroke was similar for atrial fibrillation trials (OR, 0.42;95%CI,0.35-0.51; ARR 2.5%,2 to 3) and the HFrEF trials (OR, 0.56;95%CI,0.42-0.74; ARR 1.4%,0.8 to 2) (P-interaction=0.11) (Figure 2,4). The baseline incidence of ischaemic stroke in the control group was 5.9% in atrial fibrillation trials compared to 2.9% in HFrEF trials (Figure 4).

### Oral Anticoagulation and Hemorrhagic stroke

Among sixteen studies (n=26,040), there were 79 hemorrhagic stroke events during follow-up, including 43 events in the oral anticoagulant group and 36 events in the control group ^4–6,15–22,24–27,31,37^. Oral anticoagulation compared to control was not associated with a significant increase in hemorrhagic stroke (0.29% vs 0.19% over a mean of 1.96 years) (OR, 1.23;95%CI,0.76-1.99; Absolute Risk Increase (ARI) 0.068%,0.067 to -0.2) (eFigure VI), with a consistent effect among trials (I^2^=6.0%). The association of oral anticoagulation and hemorrhagic stroke was similar for atrial fibrillation trials (OR, 1.24;95%CI,0.69-2.25; ARI 0.092%,-0.086 to 0.3) and HFrEF trials (OR, 1.20;95%CI,0.47-3.07; ARI 0.024%,0.2 to -0.18)(P-interaction=0.95) (eFigure VI).

### Oral Anticoagulation and Mortality

Among nineteen studies (n=27,771), there were 3395 deaths during follow-up including 1531 participants in the oral anticoagulant group and 1864 participants in the control group ^4,6,15,16,16–24,26–28,30–32^. Oral anticoagulation compared to control was not significantly associated with a reduction in all-cause mortality (11% vs 12% over a mean of 1.91 years) (OR, 0.95;95%CI,0.88-1.02; ARR 0.57%,-0.2 to 1.3), with a consistent effect among trials (I^2^=0%) (Figure 3, eFigure VII). There was no evidence of a statistically significant difference (I^2^=0, P-interaction=0.18), the association of oral anticoagulation and all-cause mortality was similar for atrial fibrillation trials (OR, 0.89;95%CI,0.78-1.00; ARR 0.77%,0.03 to 1.5), and HFrEF trials (OR, 0.99;95%CI,0.90-1.09; ARR 0.2%,-1 to 1.8) (Figure 2, eFigure VII). Oral anticoagulation compared to control was associated with a borderline significant reduction in cardiovascular mortality; OR, 0.90;95%CI,0.81-0.99 (eFigure VIII). The incidence of all-cause mortality in the control group was 9.6% in atrial fibrillation trials compared to 19% in HFrEF trials (Figure 3).

### Oral Anticoagulation and Myocardial Infarction

16 studies (n=27,046) reported 650 myocardial infarction events during follow-up ^4–6,15,17–20,22–24,26,27,30–32^. Oral anticoagulation compared to control was significantly associated with a reduction in myocardial infarction (2.2% vs 2.9% over a mean of 1.96 years) (OR, 0.83;95%CI,0.71-0.98; ARR 0.42%,0.06 to 0.78) (Figure 2, eFigure IX). There was no evidence of a statistically significant difference (I^2^=0%,P-interaction=0.33) between the association of oral anticoagulation and myocardial infarction, although there was a significant reduction in atrial fibrillation populations (OR, 0.76;95%CI,0.59-0.97; ARR 0.42%,0.06 to 0.79) but not HFrEF trials (OR, 0.91;95%CI,0.69-1.20; ARR 0.41%,-0.3 to 1.2) (Figure 2, eFigure IX).

### Oral Anticoagulation and Bleeding Outcomes

#### Major and Fatal hemorrhage

Among seventeen studies (n=26,474), there were 729 major hemorrhage events during follow-up ^4–6,15–22,24,26–28,30,31^. Oral anticoagulation compared with control was significantly associated with an increase in major hemorrhage (3.4% vs 2.1% over a mean of 1.85 years) (OR, 1.53;95%CI,1.08-2.16; ARI 0.85%,0.45 to 1), with an inconsistent effect among trials (I^2^=69.4%) (Figure 2, eFigure X). There was no evidence of a statistically significant difference between the association of oral anticoagulation and major hemorrhage for atrial fibrillation (OR, 1.27;95%CI,0.74-2.18; ARI 0.16%,-0.32 to 0.6) and HFrEF trials (OR, 1.91;95%CI,1.52-2.42; ARI 2.1%,1.3 to 3) (P-interaction=0.17) (Figure 2, eFigure X), or fatal hemorrhage (eFigure XI).

### Oral Anticoagulation and Original Primary Outcomes Reported in Trial

Among twenty-one studies (n=29198), there were 3823 events during follow-up including 1570 events in the oral anticoagulation group and 2253 events in the control group. For the original primary outcome, nineteen trials reported a composite outcome ^4–6,15–24,27–32^ and two reported stroke as a single primary outcome ^25,26^. Definitions of composite outcomes varied between trials (eTable I). The association of oral anticoagulation and original primary outcome differed for atrial fibrillation trials (OR, 0.58;95%CI,0.47-0.72; ARR 3.1%,2 to 3.8) and HFrEF trials (OR, 0.93;95%CI,0.85-1.02; ARR 1.3%,-0.4 to 3) (P-interaction=0.0001) (eFigure XII).

### Sensitivity Analyses

Sensitivity analysis including only trials at low risk of bias, aspirin as control, warfarin as intervention, and a targeted INR target of 2-3.5 did not materially alter the findings for all stroke, ischaemic stroke or hemorrhagic stroke and mortality (eFigure XIII, XIV, XV, XVI).

### Post-hoc Standardized Composite Outcome Measure

#### Major adverse cardiovascular events (MACE) composite outcome

Among thirteen studies (n=25,075), in which we derived major adverse cardiovascular events (MACE), oral anticoagulation compared to control was associated with a significant reduction in MACE composite outcome (OR, 0.86;95%CI,0.740-0.996; ARR 1.4%,0.6 to 2.3), with statistical evidence of heterogeneity among all trials (I^2^=65.1%,I^2^ for atrial fibrillation trials=73.8%,I^2^ for HFrEF trials=0%). The association of oral anticoagulation and MACE composite was not significant for atrial fibrillation trials (OR, 0.82;95%CI,0.63-1.07; ARR 1.4%,0.5 to 2.3) and HFrEF trials (OR, 0.92;95%CI,0.84-1.01; ARR 1.4%,-0.2 to 3.1) (P-interaction=0.43) (eFigure XVII).

In HFrEF studies (n=5) ^4–6,31,32^, oral anticoagulation compared to control was associated with a significant reduction in non-fatal cardiovascular events (OR, 0.75;95%CI,0.62-0.89, I^2^=0%) (eFigure XVIII).

## Discussion

This systematic review and meta-analysis, which included 21 trials with 29,198 participants for the primary outcome analysis, found that the association of oral anticoagulation with stroke risk is consistent for patients with atrial fibrillation and HFrEF in sinus rhythm. We found no evidence of statistically significant differences in the association of oral anticoagulation with ischaemic stroke, hemorrhagic stroke, myocardial infarction or fatal bleeding between populations. There were differences in incidence of clinical events between atrial fibrillation and HFrEF populations, with markedly higher mortality in HFrEF trials.

This updated meta-analysis extends findings from previous meta-analyses that have examined both populations separately, by including a larger number of randomised clinical trials including a recently published study ^30^ and providing comparative estimates of treatment effects in atrial fibrillation and HFrEF populations within a combined meta-analysis. While previous meta-analyses specific to trials of atrial fibrillation or HFrEF have reported similar estimates to the current analysis ^38–41^, this meta-analysis considered all trials together, which allowed us to determine the homogeneity of treatment effects across both populations. We believe that our meta-analysis provides an innovative perspective through including clinical trials from both populations in a single analysis, lending insights that may not be apparent through indirect comparisons of individual meta-analyses. Moreover, it permitted us to explore the effect of methodological differences (e.g. outcome measures) between trials of patients with atrial fibrillation and those with HFrEF. We observed a major difference in the primary outcome measures employed in trials of patients with atrial fibrillation, versus HFrEF, which have likely contributed to differing conclusions of the relative efficacy of oral anticoagulation. A key difference is that atrial fibrillation trials more often prioritised stroke in primary outcome measures (Table 1). We observed an identical magnitude of association of oral anticoagulation and stroke risk in trials of atrial fibrillation and HFrEF, which permits speculation that current guideline recommendations may be different if trials of HFrEF employed the same primary outcome measure as atrial fibrillation trials.

All HFrEF trials included mortality in a primary composite outcome measure, compared to only one atrial fibrillation trial ^16^. Although we did not identify a statistically significant difference in the association between oral anticoagulation and mortality or cardiovascular mortality (Figure 3, eFigure VII-VIII), we report a reduced risk of cardiovascular mortality in atrial fibrillation, but no significant association in HFrEF trials. Our findings suggest, but do not confirm, that there may be differing contributions of thromboembolic causes of death between these two populations. Moreover, the mortality rate in the HFrEF trials was considerably higher than in the atrial fibrillation trials, which further diluted the treatment effect on non-fatal cardiovascular events. Use of composite outcome measures in randomized clinical trials has been challenged in recent years, with the emergence of criteria for valid composite outcomes based on consistency of patient-reported importance and expected consistency in frequency of treatment effect across individual components of composite outcomes ^42^. While all-cause mortality is of major clinical importance, it appears to be unaffected by oral anticoagulation in patients with HFrEF, and would not satisfy this criterion for inclusion in a valid composite outcome ^43,44^.

Antithrombotic cardiovascular guidelines offer diverse recommendations in HFrEF, ranging from weak recommendations in HFrEF with moderate - high stroke risk ^45^, to weak recommendations against oral anticoagulant therapy ^8,46^ (eTable VI). The case for prescribing oral anticoagulation in patients with HFrEF rests on the significant reduction in stroke incidence, and clinical decision-making for individual patients will depend on the absolute risk of stroke. In contrast to atrial fibrillation (e.g. CHA_2_DS_2_-VASc score), there is no widely used score to quantify the risk of stroke in patients with HFrEF, but use of oral anticoagulation in obviously high-risk populations (e.g. prior cardioembolism) would be supported by our findings, provided the competing risk of major bleeding is acceptable. Further evidence evaluating oral anticoagulation in RCTs in HFrEF population is warranted, particularly for primary prevention where there is more uncertainty regarding risk-benefit. Based on our findings, use of oral anticoagulation would appear reasonable among patient populations who are considered at high risk of cardioembolic stroke, such as those with recent ischaemic stroke or transient ischaemic attack, where HFrEF is implicated as causal. Although the risk of bleeding in patients with HFrEF is a commonly cited reason to avoid oral anticoagulation ^40^, our findings did not reveal major differences in the risk of major or fatal bleeding in patients with atrial fibrillation compared to those with HFrEF (p for interaction=0.17). Although we note that oral anticoagulation was associated with significant increase in major haemorrhage in the HFrEF population. Anticoagulation was not associated with a significant increase in fatal haemorrhage in either population.

For many stroke physicians, anticoagulation decision-making for patients with atrial fibrillation is clear, while the decision in patients with heart failure is more challenging because of inconsistent guideline recommendations ^7,8^. Our analysis offers relevant context for the benefit of oral anticoagulation in HFrEF by comparing directly to a population where the benefit is universally accepted and consistently recommended in guidelines^1^. Clinical context (e.g. prior ischemic stroke), and patient preference, should determine selective prescribing of low-dose or treatment-dose regimens of oral anticoagulant therapy in patients with HFrEF.

### Limitations of our study

This study has several limitations. First, intervention groups consisted of a combination of two different types of oral anticoagulant therapy, vitamin K antagonists and factor Xa inhibitors, and at differing intensities including low dose rivaroxaban in the COMPASS and COMMANDER HF trials ^6,30^). We note that there are a smaller number of trials evaluating DOACs than vitamin K antagonists in HFrEF, which may warrant further investigation. Second, the INR targets varied between studies, although a sensitivity analysis restricting INR target (2-3.5), demonstrated similar results. Third, definitions of both original primary outcomes of individual trials and major hemorrhage varied among studies. This limitation is expected to be most problematic when comparing absolute rates of bleeding among trials. We have included the definition of major haemorrhage adopted by each trial in a supplementary table for clarity. Fourth, there were low event rates for some outcomes (e.g. hemorrhagic stroke), with consequence imprecise summary estimates. Although meta-analysis of rare events should be interpreted with caution, we completed a one over reciprocal continuity correction sensitivity analysis, which did not alter our findings results ^47^. Fifth, the number of patients included in atrial fibrillation trials were higher than in HFrEF trials, which limits the validity of some comparisons ^4,5,31^. Sixth, our primary outcome and research hypothesis related to all stroke, all other statistically significant results should be considered as secondary outcomes subject to the limitations of multiple testing.

## Conclusions

In this meta-analysis of randomized clinical trials, oral anticoagulation compared with control was significantly associated with a lower incidence of stroke in patients with atrial fibrillation or HFrEF. Differences in the primary outcomes employed by trials in HFrEF, compared to atrial fibrillation, may have contributed to differing conclusions of the relative efficacy of oral anticoagulation, particularly the inclusion of all-cause mortality in most HFrEF trials.

## Supplementary Material

Supplemental Publication Material

## Figures and Tables

**Table 1:** Primary outcome measures

Trial	Primary outcome
**Atrial fibrillation trials**	
AVERROES, 2011 ^15^	Occurrence of stroke (ischaemic or haemorrhagic) or systemic emboli.
WASPO, 2007 ^16^	Comparative frequency of combined endpoints comprising of death, thromboembolism, serious bleeding and withdrawal from the study.
BAFTA, 2007 ^17^	First occurrence of fatal or non-disabling stroke (ischaemic or haemorrhagic), other intracranial haemorrhage, or clinically significant arterial embolism.
Vemmos et al., 2006 ^18^	Ischaemic stroke or systemic emboli.
ACTIVE W, 2006 ^19^	First occurrence of stroke, non-CNS systemic embolism, myocardial infarction, or vascular death.
SPEAF (intermediate risk group), 2004 ^20^	Composite of vascular death, transient ischaemic attack (TIA), and nonfatal stroke or systemic embolism, whichever came first.
PATAF-stratum 1, 1999 ^21^	Stroke (ischaemic or haemorrhagic), systemic arterial embolism, major haemorrhage or vascular death.
AFASAK II, 1998 ^22^	Stroke (ischaemic or haemorrhagic) or a systemic thromboembolic event.
SPAF II (all ages), 1994 ^23^	Ischaemic stroke or systemic emboli.
EAFT, 1993 ^24^	Death from vascular disease, non-fatal stroke (including intracranial haemorrhage), non-fatal myocardial infarction, or systemic embolism.
BAATAF, 1992 ^25^	Ischaemic stroke.
Veterans, 1992 ^26^	Cerebral infarction.
SPAF, 1991 ^27^	Ischaemic stroke or systemic emboli.
CAFA, 1991 ^28^	First occurrence of any of the following: ischaemic stroke except lacunar, other systemic embolism or intracranial or fatal haemorrhage.
AFASAK, 1989 ^29^	Thromboembolic complication (TIA, minor stroke, non-disabling stroke, disabling stroke, and fatal stroke, embolism to viscera or to the extremities).
**HFrEF trials**	
COMPASS, 2019 ^30^	The primary outcome was a composite of cardiovascular death, stroke, or MI.
Commander HF, 2018 ^6^	Composite of death from any cause, myocardial infarction, or stroke.
WARCEF, 2012 ^31^	Time to first event in a composite of ischaemic stroke, intracerebral hemorrhage, or death from any cause.
WATCH, 2009 ^4^	The composite of all-cause mortality, nonfatal MI, and nonfatal stroke.
HELAS, 2006 ^32^	Any of the following: non-fatal stroke, peripheral or pulmonary embolism, myocardial (re)infarction, re-hospitalisation, exacerbation of heart failure, or death from any cause.
WASH, 2004 ^5^ 02/07/2021 13:38:00	The composite outcome of death, nonfatal myocardial infarction, and nonfatal stroke.

## Non-Standard Abbreviations and Acronyms

HFrEF: Heart failure with reduced ejection fraction
PRISMA: Preferred Reporting Items for Systematic Reviews and Meta-Analyses
REML: Restricted maximum likelihood
MACE: Major adverse cardiovascular events
RCT: Randomised clinical trials
INR: International Normalised Ratio
ARR: Absolute Risk Reduction
ARI: Absolute Risk Increase
