# Supplementary material for: Association of oral anticoagulation with stroke in atrial fibrillation or heart failure: a comparative meta-analysis
Source: Stroke. Author manuscript; Available in PMC 2021 Oct 1. (PMC8478106; doi:10.1161/STROKEAHA.120.033910)
Supplement: Supplemental Publication Material [file EMS128861-supplement-Supplemental_Publication_Material.pdf]

Supplementary Material

Table of contents

METHODS **2**

eMETHODS I – SEARCH STRATEGY 2

TABLES **3**

eTABLE I – DEFINITIONS OF COMPOSITE AND MAJOR HEMORRHAGE OUTCOMES 3
eTABLE II – TRIAL CHARACTERISTICS 5
eTABLE III – BASELINE CHARACTERISTICS 9
eTABLE IV – RISK OF BIAS SUMMARY TABLE 10
eTABLE V – PRISMA CHECKLIST 11
eTABLE VI – SUMMARY OF CURRENT GUIDELINES 12

FIGURES **13**

eFIGURE I – CONTOUR ENHANCED FUNNEL PLOT FOR ATRIAL FIBRILLATION TRIALS, ALL STROKE OUTCOME 13
eFIGURE II – CONTOUR ENHANCED FUNNEL PLOT FOR HFREF TRIALS, ALL STROKE OUTCOME 13
eFIGURE III – CROSS TABULATION RISK OF BIAS 15
eFIGURE IV – RISK OF BIAS SUMMARY 16
eFIGURE V – PRISMA FLOW DIAGRAM 17
eFIGURE VI – ORAL ANTICOAGULATION AND HEMORRHAGIC STROKE 18
eFIGURE VII – ASSOCIATION OF ORAL ANTICOAGULATION WITH ALL-CAUSE MORTALITY 19
eFIGURE VIII – ORAL ANTICOAGULATION AND CARDIOVASCULAR MORTALITY 20
eFIGURE IX – ORAL ANTICOAGULATION AND TOTAL MYOCARDIAL INFARCTION 21
eFIGURE X – ORAL ANTICOAGULATION AND MAJOR HAEMORRHAGE 22
eFIGURE XI – ORAL ANTICOAGULATION AND FATAL HEMORRHAGE 23
eFIGURE XII – ORAL ANTICOAGULATION AND ORIGINAL PRIMARY OUTCOMES OF INDIVIDUAL TRIALS 24
eFIGURE XIII – ORAL ANTICOAGULATION AND ALL STROKE - SENSITIVITY ANALYSES 25
eFIGURE XIV – ORAL ANTICOAGULATION AND ISCHAEMIC STROKE - SENSITIVITY ANALYSES 26
eFIGURE XV – ORAL ANTICOAGULATION AND HAEMORRHAGIC STROKE - SENSITIVITY ANALYSES 27
eFIGURE XVI – ORAL ANTICOAGULATION AND ALL-CAUSE MORTALITY - SENSITIVITY ANALYSES 28
eFIGURE XVII – COMPOSITE MACE OUTCOME (NON-FATAL MI, NON-FATAL STROKE, ALL-CAUSE MORTALITY) 29
eFIGURE XVIII – COMPOSITE OF NON-FATAL CARDIOVASCULAR EVENTS IN HFREF TRIALS 30

Methods

eMethods I – Search strategy

Studies were searched through November 20th, 2019.

Search terms were as follows:

Atrial fibrillation *OR* Atrial arrhythmia *OR* Heart failure
AND
Anticoagulant *OR* Anti-coagulant *OR* Anticoagulation *OR* anti thrombin *OR* Anti-thrombin *OR* Warfarin *OR* Rivaroxaban *OR* Edoxaban *OR* Apixaban *OR* Dabigatran *OR* Acenocoumarol *OR* Ximelagatran *OR* Coumarin
AND
Placebo *OR* Aspirin *OR* Clopidogrel *OR* Triflusal *OR* Ticagrelor *OR* Prasugrel
AND
Randomized controlled trial *OR* Randomised controlled trial *OR* Controlled clinical trial *OR* Randomized *OR* Randomised OR placebo *OR* Drug therapy *OR* Randomly *OR* Trial *OR* Groups
NOT
Animals *NOT* Humans

The search was conducted through Pubmed and EMBASE.

Tables

eTable I – Definitions of composite and major hemorrhage outcomes

Trial	Composite outcome	Major haemorrhage
**Atrial fibrillation trials**		
AVERROES (2011)	Occurrence of stroke (ischaemic or haemorrhagic) or systemic emboli.	Major bleeding defined as clinically overt bleeding accompanied by one or more of the following: a decrease in the haemoglobin level of 2g/dL or more over a 24 hour period, transfusion of 2 or more units of packed red cells, bleeding at a critical site (intracranial, intraspinal, intraocular, pericardial, intraarticular, intramuscular with compartment syndrome, or retroperitoneal), or fatal bleeding.
WASPO (2007)	Comparative frequency of combined endpoints comprising of death, thromboembolism, serious bleeding and withdrawal from the study.	Serious bleeding defined as intracranial haemorrhage, fall in haemoglobin by >2g/dL, need for blood transfusion.
BAFTA (2007)	First occurrence of fatal or non-disabling stroke (ischaemic or hemorrhagic), other intracranial hemorrhage, or clinically significant arterial embolism.	Major hemorrhage was defined as extracranial haemorrhage defined as a fatal haemorrhage, or one that resulted in the need for transfusion or surgery, or intracranial haemorrhage (including haemorrhagic stroke).
Vemmos et al (2006)	Ischaemic stroke or systemic emboli.	Major bleeding was defined as intracranial hemorrhage, fatal bleeding, or bleeding leading to hospital admission, emergency procedures, or urgent transfusion.
ACTIVE W (2006)	First occurrence of stroke, non-CNS systemic embolism, myocardial infarction, or vascular death.	Major bleeding was defined as any bleeding requiring transfusion of at least two units of red blood cells or equivalent of whole blood, or which was severe (severe bleeding was bleeding associated with any of the following: death, drop in haemoglobin of at least 50g/L, substantial hypotension with the need for inotropic agents, intraocular bleeding leading to substantial loss of vision, bleeding requiring surgical intervention (other than vascular site repair), symptomatic intracranial hemorrhage, or requirement for a transfusion of at least four units of blood).
SPEAF (intermediate risk group) (2004)	Composite of vascular death, transient ischaemic attack (TIA), and nonfatal stroke or systemic embolism, whichever came first.	Bleeding considered severe when requiring hospital admission, blood transfusion or surgery.
PATAF-stratum 1 (1999)	Stroke (ischaemic or hemorrhagic), systemic arterial embolism, major hemorrhage or vascular death.	Major hemorrhage defined as requiring hospital admission and blood transfusion or causing fall in haemoglobin concentration or 2.0mmol/L or more.
AFASAK II (1998)	Stroke (ischaemic or hemorrhagic) or a systemic thromboembolic event.	Major bleeding was defined as fatal, life-threatening, or potentially life-threatening, requiring surgical treatment or blood transfusion.
SPAF II (all ages) (1994)	Ischaemic stroke or systemic emboli.	Major hemorrhage was assessed by the criteria of Landefeld et al.
EAFT (1993)	Death from vascular disease, non-fatal stroke (including intracranial haemorrhage), non-fatal myocardial infarction, or systemic embolism.	Major bleeding was defined as requiring hospital admission, blood transfusion or surgery, or when it caused permanent increase in disability.
BAATAF (1992)	Not applicable.	Not applicable.
Veterans (1992)	Primary event(cerebral infarction) or death.	A major hemorrhage was defined as one that required a blood transfusion, an emergency procedure, or both to terminate bleeding or remove a hematoma or that led to admission to an intensive care unit.
SPAF (1991)	Primary event(ischaemic stroke or systemic emboli) or death.	Bleeding that involved the central nervous system, management requiring hospitalization with transfusion and/or surgery, or permanent residual impairment.
CAFA (1991)	First occurrence of any of the following: ischaemic stroke except lacunar, other systemic embolism or intracranial or fatal hemorrhage.	A major bleeding event was defined as any bleeding episode associated with 20g/L decrease or more in serum haemoglobin or requiring a blood transfusion or bleeding into a sensitive location such as the pericardium or retinal.
AFASAK (1989)	Stroke (ischaemic or hemorrhagic) or a systemic thromboembolic event.	Major bleeding was defined as requiring medical intervention.

**HFrEF trials**		
Compass (2019)	Composite of cardiovascular death, stroke, or myocardial infarction.	All bleeding that led to presentation to an acute care facility or hospitalization was considered as major. This included fatal bleeding, symptomatic bleeding into a critical organ, bleeding into a surgical site requiring reoperation, and bleeding that led to hospitalization (including presentation to an acute care facility without an overnight stay).
Commander HF (2018)	Composite of death from any cause, myocardial infarction, or stroke.	Major bleeding is defined as overt bleeding associated with a decrease in haemoglobin level of at least 2g/dL, transfusion of two or more units of packed red cells or whole blood, a critical site (intracranial, intraspinal, intraocular, pericardial, intraarticular, intramuscular with compartment syndrome or retroperitoneal), or a fatal outcome.
WARCEF (2012)	Composite end point of ischaemic stroke, intracerebral haemorrhage, or death from any cause.	Major hemorrhage was defined as intracerebral, epidural, subdural, subarachnoid, spinal intramedullary, or retinal haemorrhage; any other bleeding causing a decline in the haemoglobin level of more than 2 g per decilitre in 48 hours; or bleeding requiring transfusion of 2 or more units of whole blood, hospitalization, or surgical intervention.
WATCH (2009)	All-cause mortality, nonfatal MI and nonfatal stroke.	Major bleeding was defined as bleeding episodes leading to death or disability (including loss of neurological or special senses function), requiring surgical intervention, or associated with an acute decline of haemoglobin of greater of more than 2gm/dL or transfusion of more than 1 unit of packed red cells or whole blood.
HELAS (2006)	Non-fatal stroke, peripheral or pulmonary embolism, myocardial (re)infarction, re-hospitalisation, exacerbation of heart failure, or death from any cause.	Not reported.
WASH (2004)	The composite outcome of death, nonfatal myocardial infarction, and nonfatal stroke.	Major bleeding was defined as hemorrhage requiring transfusion.

eTable II – Trial characteristics

Trial	No. of participants	Trial design	Study Population	Intervention group	Control group	Dose/INR target	Follow-up (months)
**Atrial fibrillation trials**							
AVERROES, 2011 [15]	5599	Randomized, double-blind, double-dummy	Atrial fibrillation and ≥1 risk factor; prior stroke or TIA, >75 years, HTN, DM, HF EF <35% or PVD.	Apixaban	Aspirin	-	13.2
WASPO, 2007 [16]	75	Randomized, open label trial.	Permanent atrial fibrillation, age 80-90 years of age, ambulatory.	Warfarin	Aspirin	2-3	12
BAFTA, 2007 [17]	973	Randomized, open label trial, blinded endpoints.	Atrial fibrillation, age over 75 years.	Warfarin	Aspirin	2-3	32.4
Vemmos et al. , 2006 [18]	31	Randomized, open label trial.	Atrial fibrillation, age over 75 years.	Adjusted dose coumadin v Fixed dose coumadin	Aspirin	1.6-2.5	3.7
ACTIVE W, 2006 [19]	6706	Randomized, open label, blinded adjudication of outcomes.	Atrial fibrillation and >=1 risk factor: >75 years, HTN, previous stroke, TIA, or non-CNS systemic embolus, LV dysfunction with EF <45%,PVD.	Warfarin	Aspirin and clopidogrel	2-3	15.4

SPEAF (intermediate risk group), 2004 [20]	479	Randomized, open label trial	Chronic or documented paroxysmal AF, medium or high risk according to Stroke Prevention in Atrial Fibrillation (SPAF) III stratification.	Acenocoumarol v Triflusal and Acenocoumarol	Triflusal‡	2-3	33.1‡‡
PATAF-stratum 1, 1999 [21]††	272	Randomized, open label trial, blinded end points.	Chronic or intermittent atrial fibrillation, age over 60.	Adjusted dose coumadin v Low dose coumadin	Aspirin	2.5-3.5	32.4
AFASAK II, 1998 [22]	339	Randomized, open label trial.	Non-valvular chronic atrial fibrillation.	Minidose warfarin v Minidose warfarin and aspirin v Aspirin v Warfarin	Aspirin†	2-3	
SPAF II (all ages), 1994 [23]	1100	Randomized, open label trial.	Atrial fibrillation.	Warfarin	Aspirin	2-4.5	24
EAFT, 1993 [24]	1007	Randomized, open label oral anticoagulant, double blind aspirin or placebo trial.	Atrial fibrillation, TIA or ischaemic stroke (Rankin 3 or less) within 3 months of enrolment.	Oral anticoagulant (coumarin derivative)	Aspirin v Placebo	2.5-4	27.6
BAATAF, 1992 [25]	420	Randomized, open label trial.	Atrial fibrillation (chronic or paroxysmal).	Warfarin	Placebo**	1.5-2.7	49.2
Veterans, 1992 [26]	525	Randomized, double-blind trial	Atrial fibrillation without rheumatic heart disease	Warfarin	Placebo	1.4-2.8	20.4

SPAF, 1991 [27]	421	Randomized, open label anticoagulation arm, blinded aspirin/placebo arms.	Documented atrial fibrillation.	Warfarin	Aspirin v Placebo	2-4.5	15.6
CAFA, 1991 [28]	378	Randomized, triple blinded trial.	Atrial fibrillation (chronic or paroxysmal).	Warfarin	Placebo	2-3	15.2
AFASAK, 1989 [29]	1007	Randomized controlled trial, open label anticoagulation arm, blinded aspirin/placebo arms.	Chronic atrial fibrillation.	Warfarin	Aspirin v Placebo	2.8-4.2	24
**HFrEF trials**							
COMPASS, 2019 [30]	476	Randomized, double-blind trial.	Chronic coronary artery disease or peripheral artery disease. Subgroup- EF <40%	Rivaroxaban and Aspirin	Aspirin	-	23
Commander HF, 2018 [6]	5022	Randomized, double-blind trial.	Chronic heart failure, a left ventricular ejection fraction of 40% or less and coronary artery disease and an episode of heart failure within 21 days.	Rivaroxaban	Placebo*	-	21.1
WARCEF, 2012 [31]	2305	Randomized, double-blind, double-dummy, control trial.	Heart failure (NYHA I-IV), sinus rhythm.	Warfarin	Aspirin	2-3.5	42

WATCH, 2009 [4]	1587	Randomized control trial. Open label-warfarin, double blind-aspirin/clopidogrel.	Heart failure (NYHA II-IV), sinus rhythm.	Warfarin	Aspirin v Clopidogrel	2.5-3	22.8
HELAS, 2006 [32]	115	Randomized, double-blind, placebo control	Heart failure (NYHA II-IV), ejection fraction of less than 35%,sinus rhythm	Ischaemic heart disease<br>Warfarin	 Aspirin	2-3	18.6
	82			Dilated cardiomyopathy<br>Warfarin	 Placebo		
WASH, 2004 [5]	279	Randomized, open-label trail, blinded end point.	Heart failure (NYHA II-IV), sinus rhythm.	Warfarin	Aspirin v No antithrombotic therapy	2-3	27

*Aspirin taken by 93.1% of all patients.
†Only aspirin control arm was included in the analysis.
‡Only triflusal control arm was included in the analysis.
**Placebo group may take aspirin.
††Stratum 2 excluded from analysis as therapy evaluated was low dose anticoagulation versus aspirin.
‡‡ All values for follow-up are reported as mean with the exception of SPEAF, this follow-up value is reported as median as mean was not reported.

Abbreviations: DCM, dilated cardiomyopathy; IHD, ischaemic heart disease; TIA, transient ischaemic attack.

eTable III – Baseline characteristics

Trial	Mean Age	Female Participants, No. (%)	Subjects (Anticoagulant)	Subjects (Control)
AVERROES, 2011	70	41.5	2808	2791
WASPO, 2007	83	53.3	36	39
BAFTA, 2007	81.5	45.4	488	485
Vemmos et al. , 2006	80	49	16	15
ACTIVE W, 2006	70.2	33.9	3371	3335
SPEAF (intermediate risk group), 2004	69	43.6	237	242
PATAF-stratum 1, 1999	74.8	55.1	131	141
AFASAK II, 1998	76.5	40	170	169
SPAF II (all ages), 1994	64	24	555	545
	80	41	358	357
			197	188
EAFT, 1993	71	39.7	225	782
BAATAF, 1992	68	27.6	212	208
Veterans, 1992	67	0	260	265
SPAF, 1991	67	29	210	211
CAFA, 1991	67.7	25.4	187	191
AFASAK, 1989	74.2	46.3	335	672
**Heart failure trials**				
COMPASS, 2019	65.5	23.2	236	240
Commander HF, 2018	66.4	22.9	2507	2515
WARCEF, 2012	61	19.9	1142	1163
WATCH, 2009	63	15	540	1047
HELAS, 2006	61.7	11	54	61
	55.5	22	38	44
WASH, 2004	62.6	25.7	89	190

eTable IV – Risk of Bias Summary Table

Trial	Randomization process	Deviations from intended interventions	Missing outcome data	Measurement of the outcome	Selection of the reported result	Overall Bias
**Atrial Fibrillation Trials**						
AVERROES (2011)	Low	Low	Low	Low	Low	Low
WASPO (2007)	Low	Low	Low	Low	Low	Low
BAFTA (2007)	Low	Low	Low	Low	Low	Low
Vemmos et al (2006)	Some Concerns	Low	Low	Some Concerns	Low	Some Concerns
ACTIVE W (2006)	Low	Low	Low	Low	Low	Low
SPEAF (2004)	Low	High	Low	Low	Low	High
PATAF (1999)	Low	Low	Low	Low	Low	Low
AFASAK II (1998)	Low	Low	Low	Low	Low	Low
SPAF II (1994)	Low	Low	Low	Low	Low	Low
EAFT (1993)	Low	Low	Low	Low	Low	Low
BAATAF (1992)	Some Concerns	High	Low	Low	Low	High
Veterans (1992)	Low	Low	Low	Low	Low	Low
SPAF (1991)	Low	Low	Low	Low	Low	Low
CAFA (1991)	Some Concerns	Low	Some Concerns	Low	Low	Some Concerns
AFASAK (1989)	Low	Low	Low	High	Low	High
**HFrEF**						
COMPASS (2019)	Low	Low	Low	Low	Low	Low
COMMANDER HF (2018)	Low	Low	Low	Low	Low	Low
WARCEF (2012)	Low	Low	Low	Low	Low	Low
WATCH (2009)	Low	Low	Low	Low	Low	Low
HELAS (2006)	Low	Some Concerns	Low	Low	Low	Some Concerns
WASH (2004)	Low	Low	Low	Low	Low	Low

eTable V – Prisma checklist

Section/topic	#	Checklist item	Reported on page #
**TITLE**			
Title	1	Identify the report as a systematic review, meta-analysis, or both.	1
**ABSTRACT**			
Structured summary	2	Provide a structured summary including, as applicable: background; objectives; data sources; study eligibility criteria, participants, and interventions; study appraisal and synthesis methods; results; limitations; conclusions and implications of key findings; systematic review registration number.	3-4
**INTRODUCTION**			
Rationale	3	Describe the rationale for the review in the context of what is already known.	5
Objectives	4	Provide an explicit statement of questions being addressed with reference to participants, interventions, comparisons, outcomes, and study design (PICOS).	6-7
**METHODS**			
Protocol and registration	5	Indicate if a review protocol exists, if and where it can be accessed (e.g., Web address), and, if available, provide registration information including registration number.	(PROSPERO identifier: CRD42020153013)
Eligibility criteria	6	Specify study characteristics (e.g., PICOS, length of follow-up) and report characteristics (e.g., years considered, language, publication status) used as criteria for eligibility, giving rationale.	7
Information sources	7	Describe all information sources (e.g., databases with dates of coverage, contact with study authors to identify additional studies) in the search and date last searched.	7
Search	8	Present full electronic search strategy for at least one database, including any limits used, such that it could be repeated.	7
Study selection	9	State the process for selecting studies (i.e., screening, eligibility, included in systematic review, and, if applicable, included in the meta-analysis).	7
Data collection process	10	Describe method of data extraction from reports (e.g., piloted forms, independently, in duplicate) and any processes for obtaining and confirming data from investigators.	8
Data items	11	List and define all variables for which data were sought (e.g., PICOS, funding sources) and any assumptions and simplifications made.	8
Risk of bias in individual studies	12	Describe methods used for assessing risk of bias of individual studies (including specification of whether this was done at the study or outcome level), and how this information is to be used in any data synthesis.	9
Summary measures	13	State the principal summary measures (e.g., risk ratio, difference in means).	9
Synthesis of results	14	Describe the methods of handling data and combining results of studies, if done, including measures of consistency (e.g., I^2) for each meta-analysis.	8-9

eTable VI – Summary of current guidelines

Guideline	Atrial fibrillation (Year)	HFrEF (Year)
**European Society of Cardiology**	Grade 1A recommendation for oral anticoagulation to prevent thromboembolism in those with a $CHA_2DS_2\text{-}VAS_C \geq 2$ (2020) [1].	Grade IIa recommendation for chronic cardiac syndrome in sinus rhythm at high stroke risk (IIb for moderate risk) to consider dual antiplatelet therapy or low dose rivaroxaban (2019) [45].
**American Heart Association**	Class 1 recommendation for oral anticoagulation for primary stroke prevention (warfarin 1A, DOAC 1B) (2019) [2].	Do not recommend anticoagulation in patients with heart failure with reduced ejection fraction in the absence of atrial fibrillation (level B) (2013) [8].
**Canadian Cardiovascular Society**	Recommend anticoagulation for those with a $CHA_2DS_2\text{-}VAS_C \geq 1$ (2018) [3]	
**The American College of Chest Physicians**		Recommend anticoagulation for a three-month period following an anterior myocardial infarction and left ventricular thrombus or an ejection fraction of ≤40%. Grade 2C recommendation against anticoagulation for those with non-ischaemic LV dysfunction (2012) [46].
**Heart failure society of America**		Treatment with warfarin (goal INR 2.0-3.0) is recommended for all patients with HF and a history of systemic or pulmonary emboli, including stroke or transient ischaemic attack, (Strength of Evidence C) (2010) [7].

Figures

eFigure I – Contour enhanced funnel plot for atrial fibrillation trials, all stroke outcome

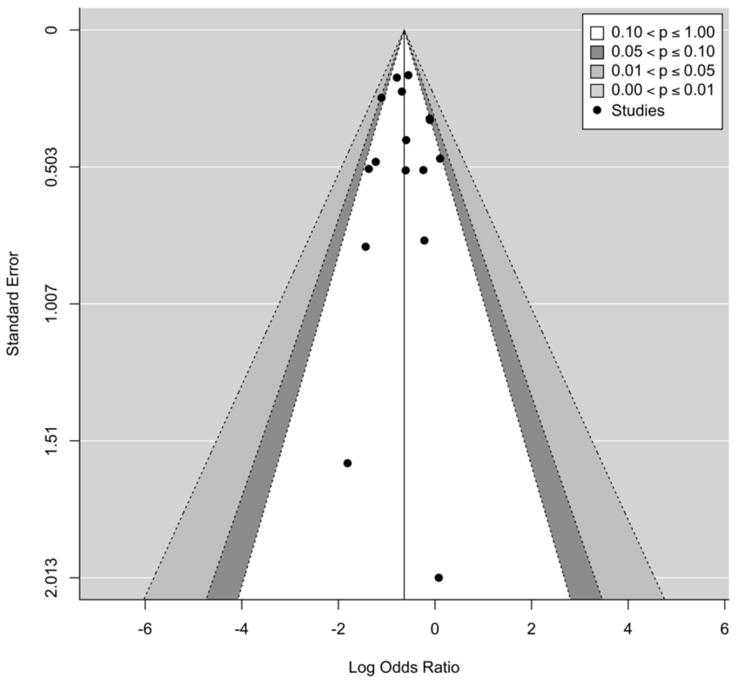

eFigure I – Contour enhanced funnel plot for atrial fibrillation trials, all stroke outcome. Different levels of statistical significance for studies are indicated by the shaded regions, detailed within the figure. The grey vertical line represents the summary estimate for oral anticoagulation and all stroke.

eFigure II – Contour enhanced funnel plot for HFrEF trials, all stroke outcome

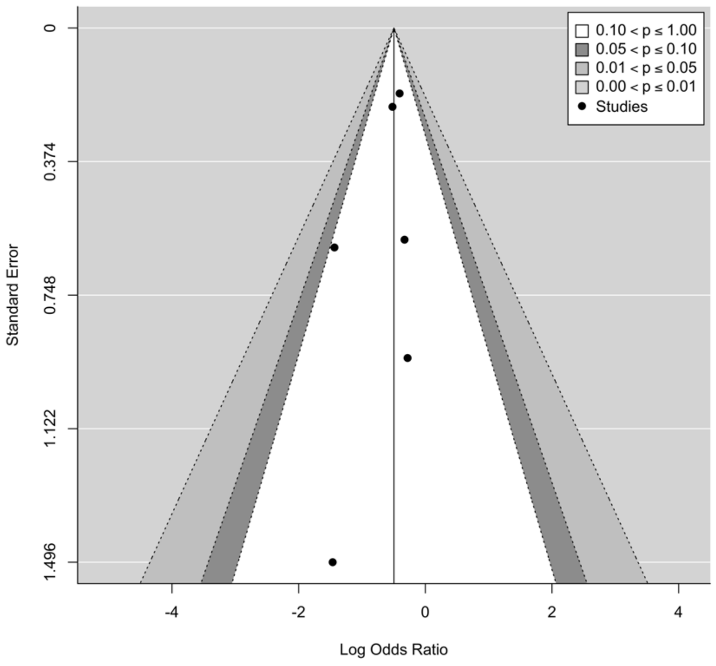

eFigure II – Contour enhanced funnel plot for HFrEF trials, all stroke outcome. Different levels of statistical significance for studies are indicated by the shaded regions, detailed within the figure. The grey vertical line represents the summary estimate for oral anticoagulation and all stroke.

eFigure III – Cross Tabulation Risk of Bias

	Unique ID	Outcome	Weight	Randomization process	Deviations from intended interventions	Missing outcome data	Measurement of the outcome	Selection of the reported result	Overall
1	WARCEF	All stroke	9.79	+	+	+	+	+	+
2	WATCH	All stroke	1.61	+	+	+	+	+	+
3	WASH	All stroke	0.28	+	+	+	+	+	+
4	WASPO	All stroke	0.16	+	+	+	+	+	+
5	BAFTA	All stroke	9.43	+	+	+	+	+	+
6	AFASAK II	All stroke	2.64	+	+	+	+	+	+
7	ACTIVE W	All stroke	14.57	+	+	+	+	+	+
8	SPAF II	All stroke	10.13	+	+	+	+	+	+
9	AVERROES	All stroke	13.6	+	+	+	+	+	+
10	EAFT	All stroke	8.11	+	+	+	+	+	+
11	COMMANDER HF	All stroke	12.8	+	+	+	+	+	+
12	AFASAK	All stroke	2.53	+	+	+	–	+	–
13	HELAS	All stroke	0.73	+	?	+	+	+	!
14	SPEAF	All stroke	2.25	+	–	+	+	+	–
15	BAATAF	All stroke	1.05	?	–	+	+	+	–
16	SPAF	All stroke	2.78	+	+	+	+	+	+
17	Vemmos et al	All stroke	0.25	?	+	+	?	+	!
18	PATAF	All stroke	1.03	+	+	+	+	+	+
19	Veterans	All stroke	2.29	+	+	+	+	+	+
20	CAFA	All stroke	2.26	?	+	+	+	+	!
21	COMPASS	All stroke	1.72	+	+	+	+	+	+

\+ Low risk
? Some concerns
– High risk

eFigure III was generated using the Cochrane Risk of Bias 2 tool.
https://methods.cochrane.org/bias/resources/rob-2-revised-cochrane-risk-bias-tool-randomized-trials

eFigure IV – Risk of Bias Summary

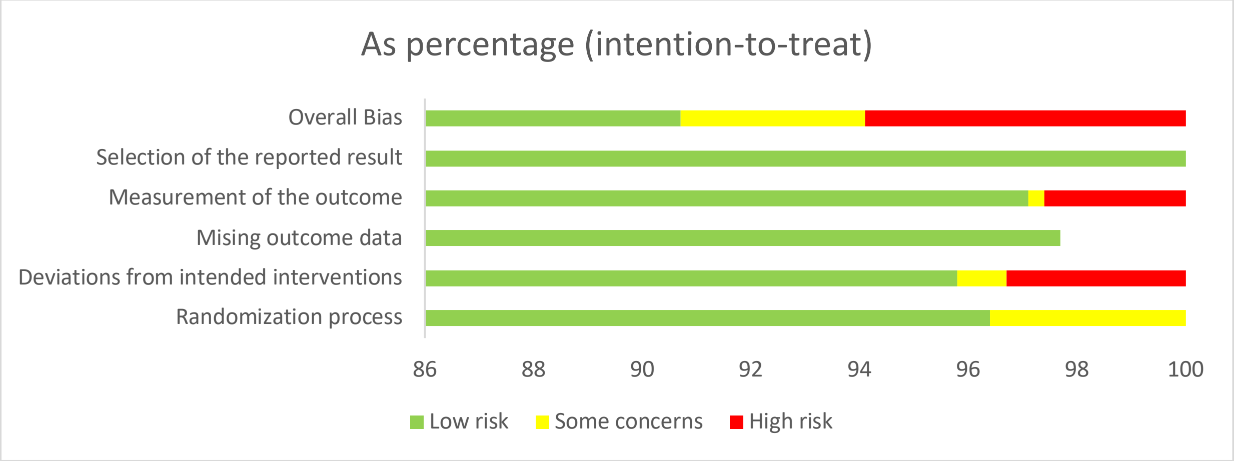

eFigure IV was generating using the Cochrane Risk of Bias 2 tool.
https://methods.cochrane.org/bias/resources/rob-2-revised-cochrane-risk-bias-tool-randomized-trials

eFigure V – Prisma flow diagram

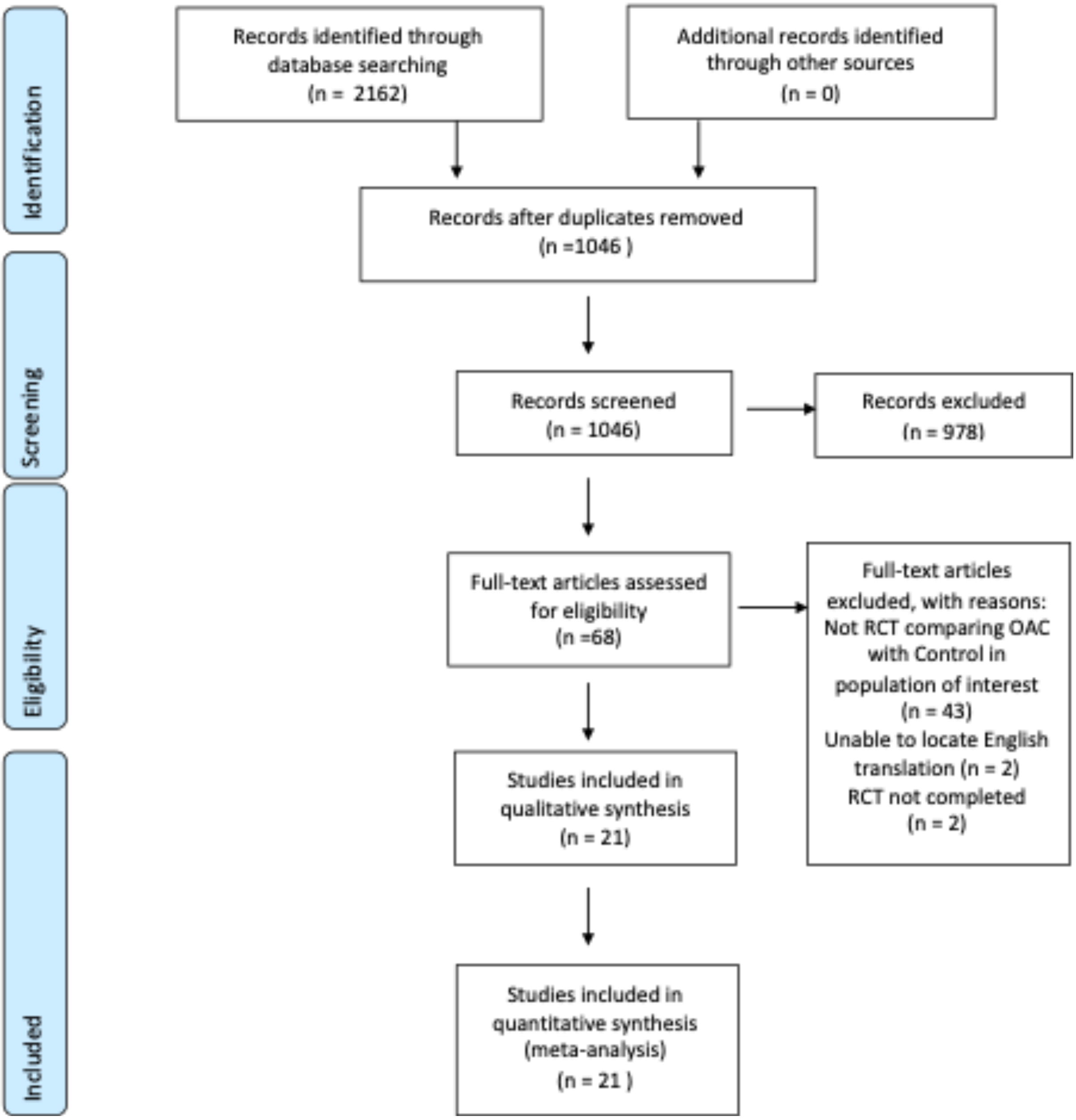

eFigure VI – Oral anticoagulation and Hemorrhagic stroke

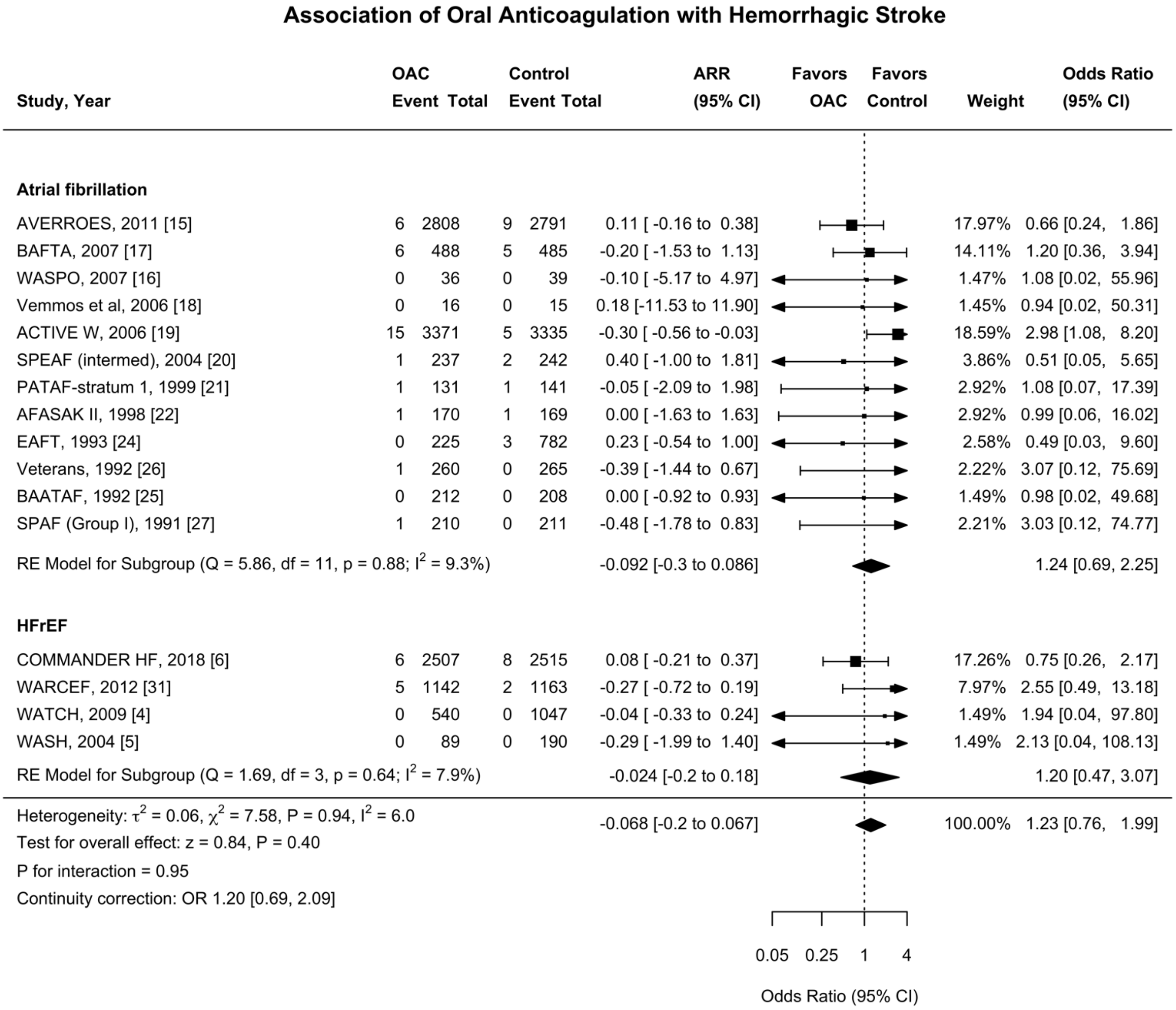

eFigure VI – Forest plot demonstrating the association of oral anticoagulant with hemorrhagic stroke events compared to control. The squares and bars represent the mean values and 95% confidence intervals of the effect sizes, while the size of the squares reflects the weight of the studies. The combined effects appear as diamonds and the vertical dashed line represents the line of no effect. Int-Intervention, CI-Confidence Interval, ARR-Absolute risk reduction.

eFigure VII – Association of Oral Anticoagulation with All-cause Mortality

Association of Oral Anticoagulation with All-cause Mortality

Study, Year	Int Event	Int Total	Control Event	Control Total	ARR (95% CI)	Weight	Odds Ratio (95% CI)
**Atrial fibrillation**							
AVERROES, 2011 [15]	111	2808	140	2791	1.06 [-0.02 to 2.1]	8.85%	0.78 [0.60, 1.01]
BAFTA, 2007 [17]	107	488	108	485	0.34 [-4.87 to 5.6]	6.27%	0.98 [0.72, 1.33]
WASPO, 2007 [16]	1	36	2	39	2.35 [-6.41 to 11.1]	0.10%	0.53 [0.05, 6.09]
Vemmos et al, 2006 [18]	0	16	0	15	0.18 [-11.53 to 11.9]	0.04%	0.94 [0.02, 50.31]
ACTIVE W, 2006 [19]	158	3371	159	3335	0.08 [-0.94 to 1.1]	11.30%	0.98 [0.78, 1.23]
SPEAF (intermed), 2004 [20]	20	237	15	242	-2.24 [-6.90 to 2.4]	1.19%	1.39 [0.70, 2.79]
PATAF-stratum 1, 1999 [21]	12	131	17	141	2.90 [-4.40 to 10.2]	0.94%	0.74 [0.34, 1.61]
AFASAK II, 1998 [22]	17	170	14	169	-1.72 [-7.85 to 4.4]	1.04%	1.23 [0.59, 2.58]
SPAF II (>75), 1994 [23]	26	197	24	188	-0.43 [-7.15 to 6.3]	1.63%	1.04 [0.57, 1.88]
SPAF II (<75), 1994 [23]	36	358	41	357	1.43 [-3.11 to 6.0]	2.56%	0.86 [0.54, 1.38]
EAFT, 1993 [24]	41	225	201	782	7.48 [1.58 to 13.4]	4.10%	0.64 [0.44, 0.94]
Veterans, 1992 [26]	15	260	22	265	2.53 [-1.83 to 6.9]	1.24%	0.68 [0.34, 1.33]
SPAF (Group I), 1991 [27]	6	210	8	211	0.93 [-2.49 to 4.4]	0.50%	0.75 [0.25, 2.19]
CAFA, 1991 [28]	10	187	8	191	-1.16 [-5.46 to 3.1]	0.63%	1.29 [0.50, 3.35]
RE Model for Subgroup (Q = 9.37, df = 13, p = 0.74; I^2 = 2.9%)					0.77 [0.03 to 1.5]		0.89 [0.78, 1.00]
**Heart failure**							
COMPASS, 2019 [30]	23	236	24	240	0.25 [-5.11 to 5.6]	1.58%	0.97 [0.53, 1.78]
COMMANDER HF, 2018 [6]	546	2507	556	2515	0.33 [-1.96 to 2.6]	32.18%	0.98 [0.86, 1.12]
WARCEF, 2012 [31]	275	1142	272	1163	-0.69 [-4.17 to 2.8]	15.61%	1.04 [0.86, 1.26]
WATCH, 2009 [4]	92	540	190	1047	1.11 [-2.83 to 5.0]	7.66%	0.93 [0.70, 1.22]
HELAS, 2006 [32]	13	92	15	105	0.16 [-9.62 to 9.9]	0.89%	0.99 [0.44, 2.20]
WASH, 2004 [5]	22	89	48	190	0.54 [-10.34 to 11.4]	1.70%	0.97 [0.54, 1.74]
RE Model for Subgroup (Q = 0.49, df = 5, p = 0.99; I^2 = 0.0%)					0.2 [-1 to 1.8]		0.99 [0.90, 1.09]
Heterogeneity: τ^2 = 0.00, χ^2 = 11.76, P = 0.90, I^2 = 0.0					0.57 [-0.2 to 1.3]	100.00%	0.95 [0.88, 1.02]

Test for overall effect: z = -1.44, P = 0.15

P for interaction = 0.18

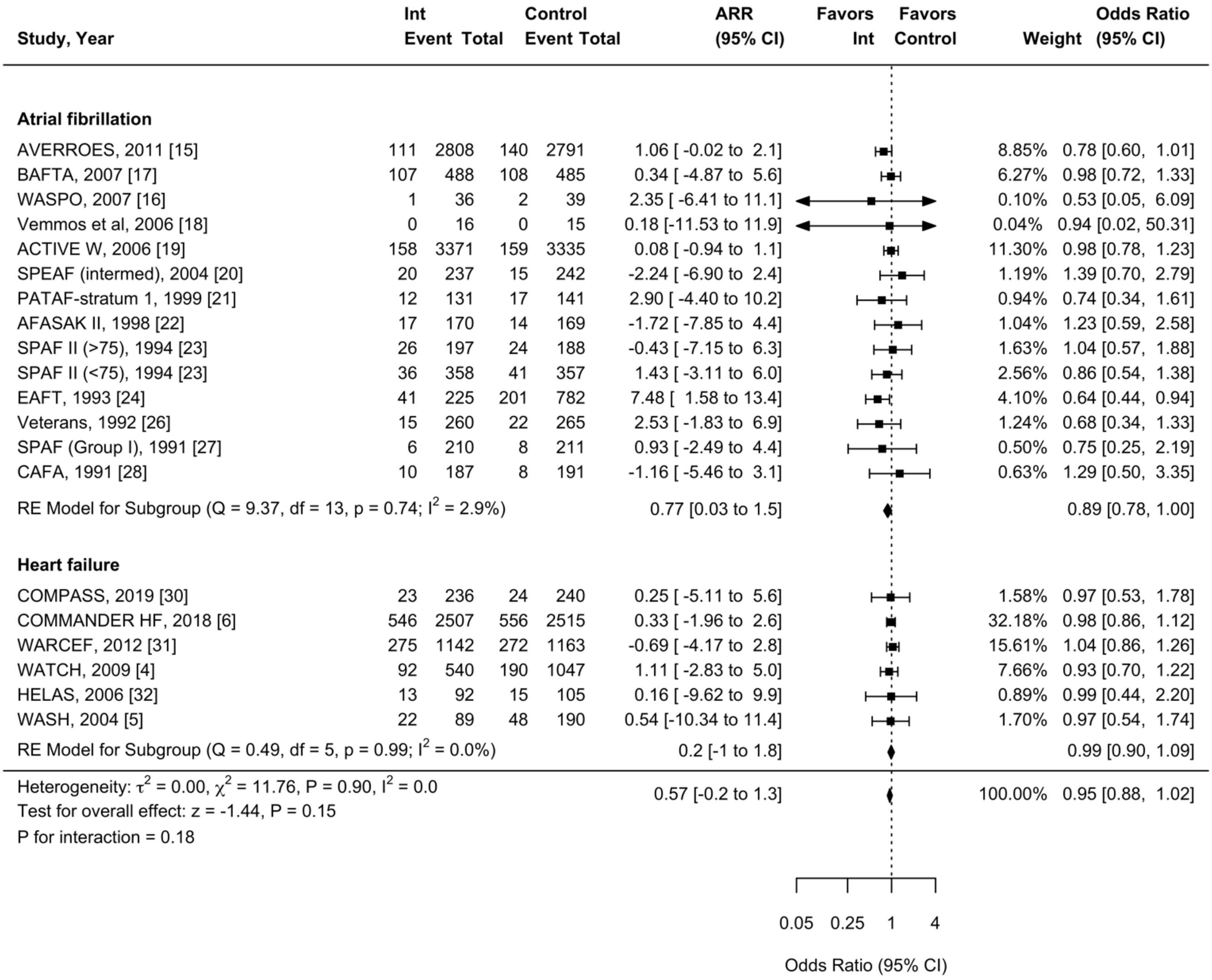

eFigure VII – Forest plot demonstrating the association of oral anticoagulant with all-cause mortality events compared to control. The squares and bars represent the mean values and 95% confidence intervals of the effect sizes, while the size of the squares reflects the weight of the studies. The combined effects appear as diamonds and the vertical dashed line represents the line of no effect. Int-Intervention, CI-Confidence Interval, ARR-Absolute risk reduction.

eFigure VIII – Oral anticoagulation and Cardiovascular Mortality

Association of Oral Anticoagulation with Cardiovascular Mortality

Study, Year	Int Event	Int Total	Control Event	Control Total	ARR (95% CI)	Weight	Odds Ratio (95% CI)
**Atrial fibrillation**							
AVERROES, 2011 [15]	84	2808	96	2791	0.45 [-0.48 to 1.4]	11.46%	0.87 [0.64, 1.17]
BAFTA, 2007 [17]	56	488	57	485	0.28 [-3.75 to 4.3]	6.60%	0.97 [0.66, 1.44]
Vemmos et al, 2006 [18]	0	16	0	15	0.18 [-11.53 to 11.9]	0.06%	0.94 [0.02, 50.31]
ACTIVE W, 2006 [19]	106	3371	120	3335	0.45 [-0.41 to 1.3]	14.37%	0.87 [0.67, 1.13]
SPEAF (intermed), 2004 [20]	11	237	8	242	-1.34 [-4.84 to 2.2]	1.18%	1.42 [0.56, 3.60]
PATAF-stratum 1, 1999 [21]	9	131	9	141	-0.49 [-6.41 to 5.4]	1.11%	1.08 [0.42, 2.81]
AFASAK II, 1998 [22]	5	170	4	169	-0.57 [-4.00 to 2.9]	0.57%	1.25 [0.33, 4.74]
SPAF II (>75), 1994 [23]	16	197	14	188	-0.68 [-6.03 to 4.7]	1.82%	1.10 [0.52, 2.32]
SPAF II (<75), 1994 [23]	20	358	25	357	1.42 [-2.14 to 5.0]	2.75%	0.79 [0.43, 1.44]
EAFT, 1993 [24]	30	225	156	782	6.62 [1.36 to 11.9]	5.69%	0.62 [0.40, 0.94]
SPAF (Group I), 1991 [27]	4	210	7	211	1.41 [-1.63 to 4.5]	0.66%	0.57 [0.16, 1.96]
CAFA, 1991 [28]	9	187	6	191	-1.67 [-5.61 to 2.3]	0.91%	1.56 [0.54, 4.47]
AFASAK, 1989 [29]	3	335	27	672	3.12 [1.33 to 4.9]	0.70%	0.22 [0.07, 0.72]
RE Model for Subgroup (Q = 11.66, df = 12, p = 0.47; I^2 = 0.0%)					0.73 [0.1 to 1.3]		0.86 [0.74, 0.99]
**Heart failure**							
COMPASS, 2019 [30]	16	236	19	240	1.14 [-3.55 to 5.8]	2.13%	0.85 [0.42, 1.69]
COMMANDER HF, 2018 [6]	453	2507	476	2515	0.86 [-1.29 to 3.0]	49.99%	0.94 [0.82, 1.09]
RE Model for Subgroup (Q = 0.09, df = 1, p = 0.76; I^2 = 0.0%)					0.88 [-1 to 2.9]		0.94 [0.82, 1.08]
Heterogeneity: τ^2 = 0.00, χ^2 = 12.61, P = 0.56, I^2 = 0.0					0.76 [0.1 to 1.4]	100.00%	0.90 [0.81, 0.99]

Test for overall effect: z = -2.08, P = 0.04

P for interaction = 0.36

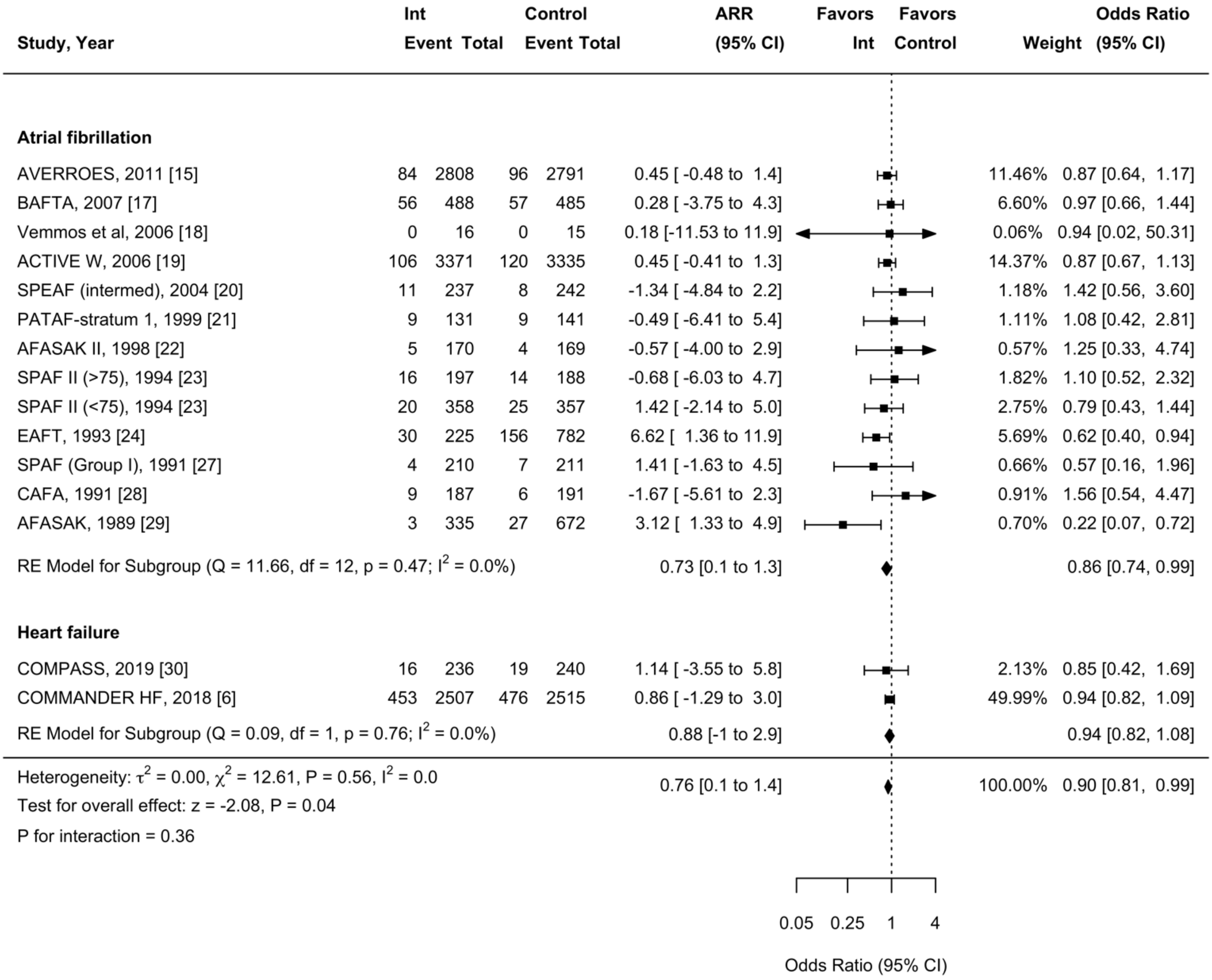

eFigure VIII – Forest plot demonstrating the association of oral anticoagulant with cardiovascular mortality events compared to control. The squares and bars represent the mean values and 95% confidence intervals of the effect sizes, while the size of the squares reflects the weight of the studies. The combined effects appear as diamonds and the vertical dashed line represents the line of no effect. Int-Intervention, CI-Confidence Interval, ARR-Absolute risk reduction.

eFigure IX – Oral anticoagulation and Total Myocardial Infarction

Association of Oral Anticoagulation with Total Myocardial Infarction

Study, Year	Int Event	Int Total	Control Event	Control Total	ARR (95% CI)	Weight	Odds Ratio (95% CI)
**Atrial fibrillation**							
AVERROES, 2011 [15]	24	2808	28	2791	0.15 [-0.35 to 0.65]	8.97%	0.85 [0.49, 1.47]
BAFTA, 2007 [17]	15	488	15	485	0.02 [-2.15 to 2.19]	5.09%	0.99 [0.48, 2.06]
Vemmos et al, 2006 [18]	0	16	0	15	0.18 [-11.53 to 11.90]	0.17%	0.94 [0.02, 50.31]
ACTIVE W, 2006 [19]	23	3371	36	3335	0.40 [-0.05 to 0.84]	9.75%	0.63 [0.37, 1.06]
SPEAF (intermed), 2004 [20]	0	237	1	242	0.41 [-0.74 to 1.55]	0.26%	0.34 [0.01, 8.36]
AFASAK II, 1998 [22]	4	170	4	169	0.01 [-3.22 to 3.25]	1.37%	0.99 [0.24, 4.04]
SPAF II (>75), 1994 [23]	5	197	5	188	0.12 [-3.06 to 3.30]	1.71%	0.95 [0.27, 3.35]
SPAF II (<75), 1994 [23]	10	358	14	357	1.13 [-1.51 to 3.77]	3.95%	0.70 [0.31, 1.61]
EAFT, 1993 [24]	17	225	81	782	2.80 [-1.26 to 6.86]	9.05%	0.71 [0.41, 1.22]
Veterans, 1992 [26]	1	260	4	265	1.12 [-0.52 to 2.77]	0.56%	0.25 [0.03, 2.27]
SPAF (Group I), 1991 [27]	2	210	2	211	0.00 [-1.86 to 1.85]	0.69%	1.00 [0.14, 7.20]
RE Model for Subgroup (Q = 2.84, df = 10, p = 0.98; I^2 = 0.0%)					0.42 [0.06 to 0.79]		0.76 [0.59, 0.97]
**Heart failure**							
COMPASS, 2019 [30]	6	236	10	240	1.62 [-1.60 to 4.85]	2.54%	0.60 [0.21, 1.68]
COMMANDER HF, 2018 [6]	98	2507	118	2515	0.78 [-0.34 to 1.90]	35.89%	0.83 [0.63, 1.09]
WARCEF, 2012 [31]	28	1142	31	1163	0.21 [-1.08 to 1.50]	10.04%	0.92 [0.55, 1.54]
WATCH, 2009 [4] *	21	540	27	1047	-1.31 [-3.20 to 0.58]	8.00%	1.53 [0.86, 2.73]
HELAS, 2006 [32]	2	92	0	105	-2.22 [-5.75 to 1.32]	0.29%	5.83 [0.28, 122.99]
WASH, 2004 [5]	3	89	15	190	4.52 [-0.84 to 9.89]	1.68%	0.41 [0.11, 1.44]
RE Model for Subgroup (Q = 7.13, df = 5, p = 0.21; I^2 = 16.2%)					0.41 [-0.3 to 1.2]		0.91 [0.69, 1.20]
Heterogeneity: τ^2 = 0.00, χ^2 = 10.94, P = 0.81, I^2 = 0.0					0.42 [0.06 to 0.78]	100.00%	0.83 [0.71, 0.98]

Test for overall effect: z = -2.18, P = 0.03

P for interaction = 0.33

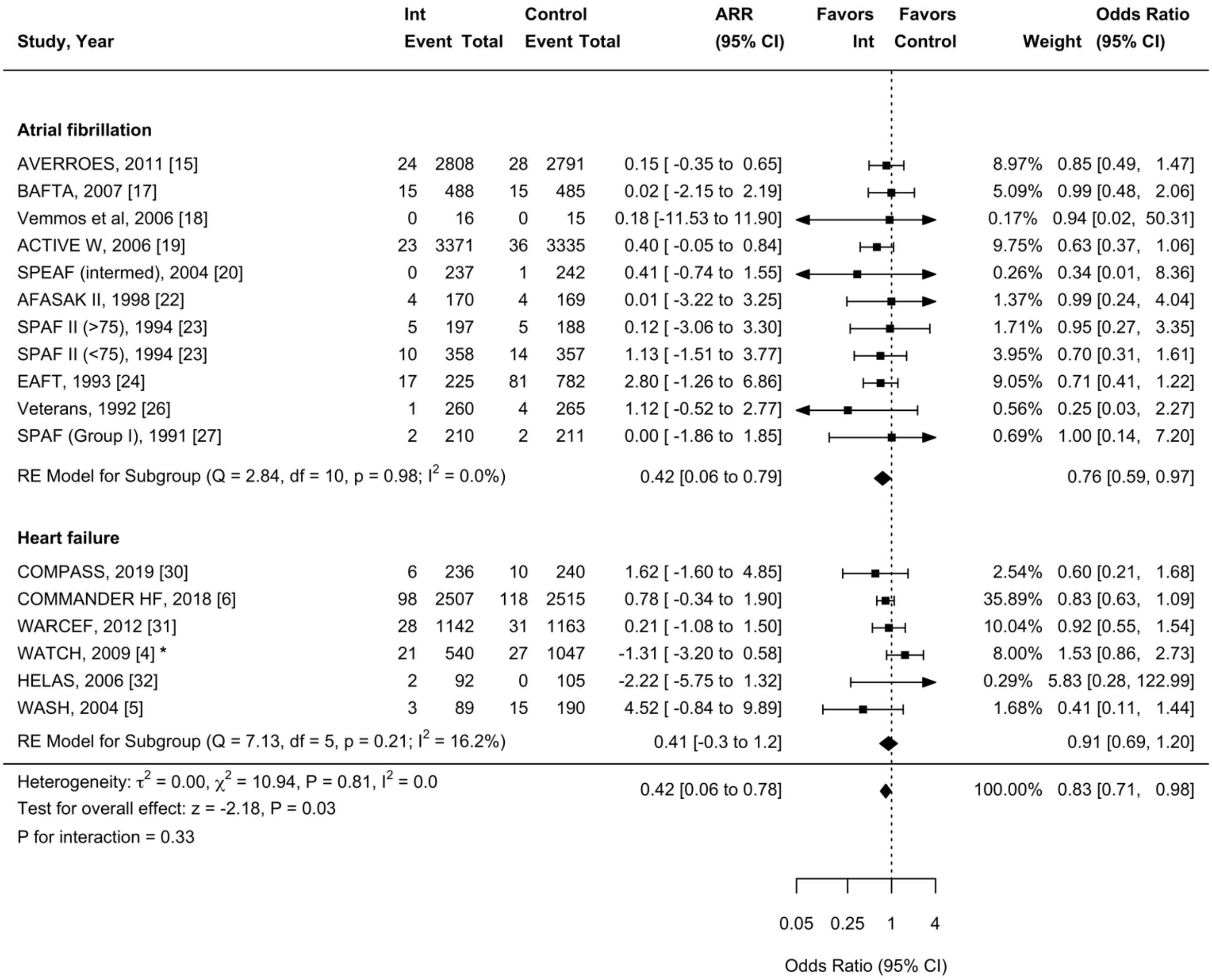

eFigure IX – Forest plot demonstrating the association of oral anticoagulant with total myocardial infarction events compared to control. The squares and bars represent the mean values and 95% confidence intervals of the effect sizes, while the size of the squares reflects the weight of the studies. The combined effects appear as diamonds and the vertical dashed line represents the line of no effect. Int-Intervention, CI-Confidence Interval, ARR-Absolute risk reduction, MI-Myocardial Infarction.

*WATCH reports non-fatal MI.

eFigure X – Oral anticoagulation and Major Haemorrhage

Association of Oral Anticoagulation with Major Hemorrhage

Study, Year	Int Event	Int Total	Control Event	Control Total	ARR (95% CI)	Weight	Odds Ratio (95% CI)
**Atrial fibrillation**							
AVERROES, 2011 [15]	44	2808	39	2791	-0.17 [-0.80 to 0.46]	10.23%	1.12 [0.73, 1.73]
BAFTA, 2007 [17]	25	488	25	485	0.03 [-2.74 to 2.81]	9.18%	0.99 [0.56, 1.76]
WASPO, 2007 [16]	0	36	3	39	7.40 [-2.12 to 16.91]	1.21%	0.14 [0.01, 2.87]
Vemmos et al, 2006 [18]	0	16	0	15	0.18 [-11.53 to 11.90]	0.72%	0.94 [0.02, 50.31]
ACTIVE W, 2006 [19]	93	3371	101	3335	0.27 [-0.53 to 1.07]	11.25%	0.91 [0.68, 1.21]
SPEAF (intermed), 2004 [20]	10	237	2	242	-3.39 [-6.20 to -0.59]	3.63%	5.29 [1.15, 24.39]
PATAF-stratum 1, 1999 [21]	2	131	1	141	-0.82 [-3.33 to 1.70]	1.78%	2.17 [0.19, 24.22]
AFASAK II, 1998 [22]	4	170	5	169	0.61 [-2.82 to 4.03]	4.37%	0.79 [0.21, 3.00]
EAFT, 1993 [24]	13	225	10	782	-4.50 [-7.65 to -1.35]	7.13%	4.73 [2.05, 10.95]
Veterans, 1992 [26]	6	260	4	265	-0.80 [-3.14 to 1.54]	4.61%	1.54 [0.43, 5.53]
SPAF (Group I), 1991 [27]	4	210	14	211	4.73 [0.90 to 8.56]	5.34%	0.27 [0.09, 0.84]
CAFA, 1991 [28]	5	187	1	191	-2.15 [-4.68 to 0.38]	2.14%	5.22 [0.60, 45.11]
RE Model for Subgroup (Q = 27.93, df = 11, p = 0.00; I^2 = 74.0%)					-0.16 [-0.6 to 0.32]		1.27 [0.74, 2.18]
**Heart failure**							
COMPASS, 2019 [30]	11	236	5	240	-2.58 [-5.82 to 0.66]	5.64%	2.30 [0.79, 6.72]
COMMANDER HF, 2018 [6]	82	2507	50	2515	-1.28 [-2.17 to -0.40]	10.79%	1.67 [1.17, 2.38]
WARCEF, 2012 [31]	72	1142	35	1163	-3.30 [-5.01 to -1.58]	10.39%	2.17 [1.44, 3.28]
WATCH, 2009 [4]	28	540	30	1047	-2.32 [-4.45 to -0.19]	9.52%	1.85 [1.10, 3.14]
WASH, 2004 [5]	4	89	1	190	-3.97 [-8.39 to 0.46]	2.06%	8.89 [0.98, 80.77]
RE Model for Subgroup (Q = 2.92, df = 4, p = 0.57; I^2 = 0.0%)					-2.1 [-3 to -1.3]		1.91 [1.52, 2.42]
Heterogeneity: τ^2 = 0.26, χ^2 = 44.59, P = 0.00, I^2 = 69.4					-0.85 [-1 to -0.45]	100.00%	1.53 [1.08, 2.16]
Test for overall effect: z = 2.39, P = 0.02							
P for interaction = 0.17							

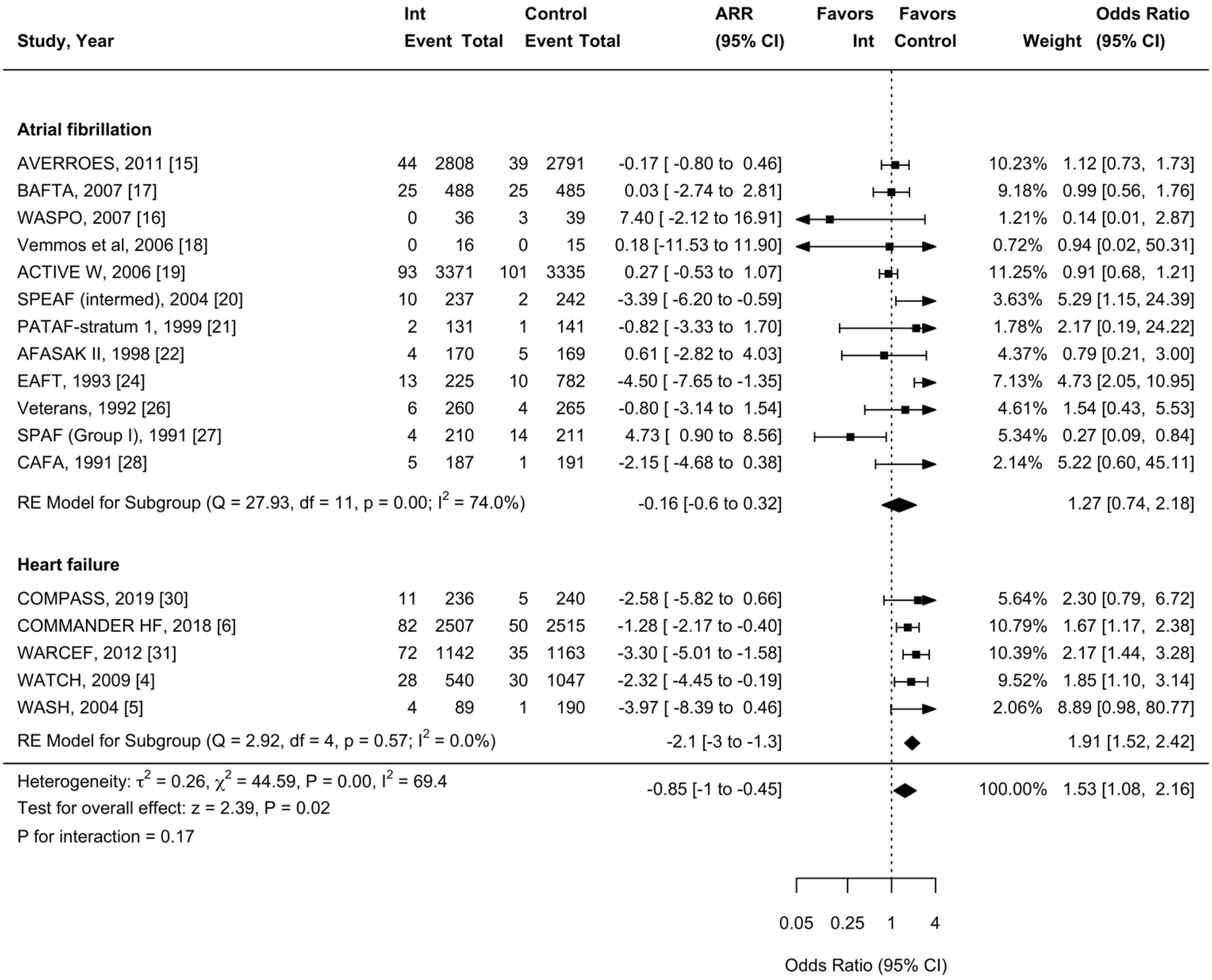

eFigure X – Forest plot demonstrating the association of oral anticoagulant with major haemorrhage events compared to control. The squares and bars represent the mean values and 95% confidence intervals of the effect sizes, while the size of the squares reflects the weight of the studies. The combined effects appear as diamonds and the vertical dashed line represents the line of no effect. Int-Intervention, CI-Confidence Interval, ARR-Absolute risk reduction.

Definitions of major haemorrhage for individual trials can be found in eTable I.

eFigure XI – Oral anticoagulation and Fatal Hemorrhage

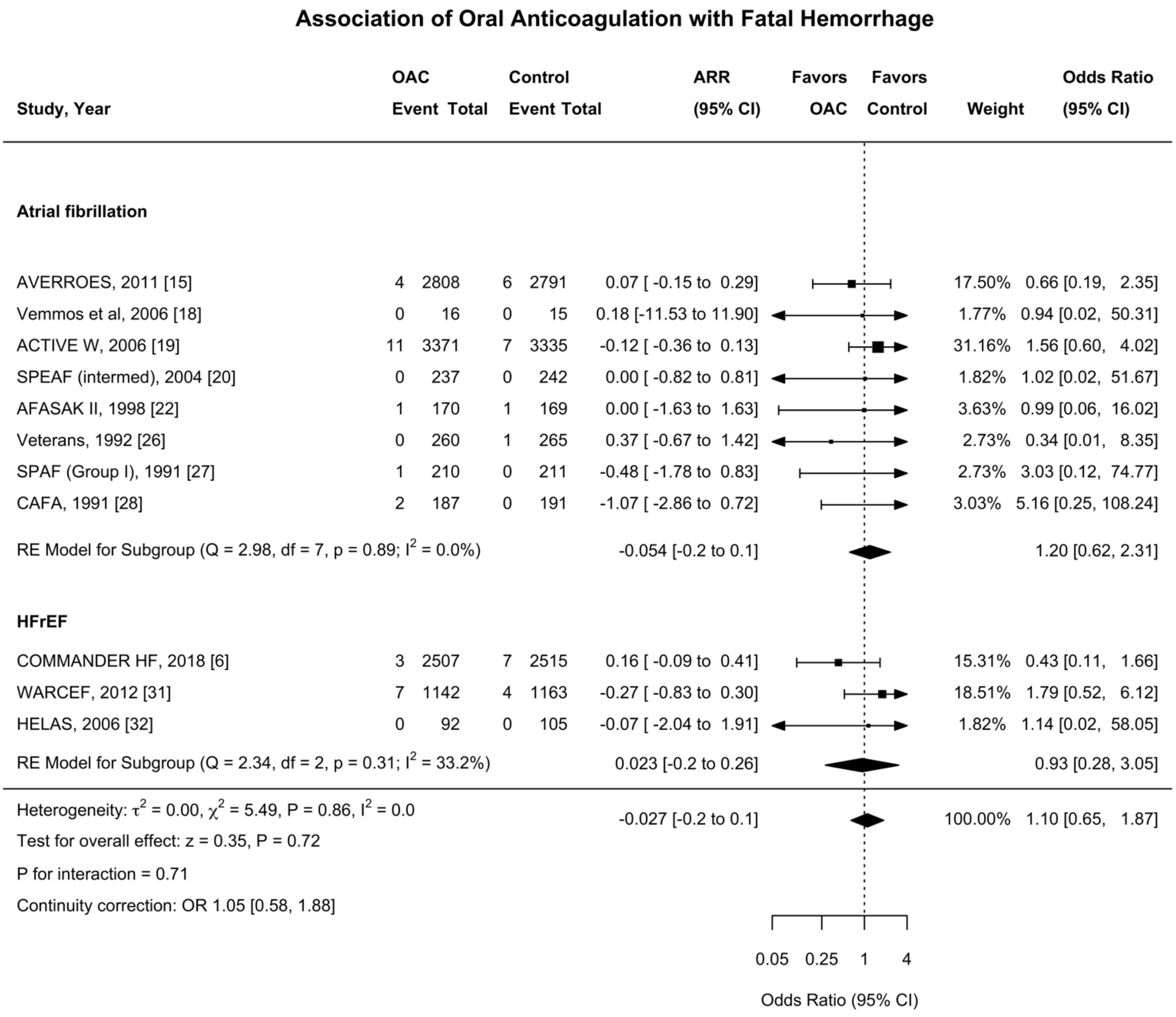

eFigure XI – Forest plot demonstrating the association of oral anticoagulant with fatal hemorrhage events compared to control. The squares and bars represent the mean values and 95% confidence intervals of the effect sizes, while the size of the squares reflects the weight of the studies. The combined effects appear as diamonds and the vertical dashed line represents the line of no effect. Int-Intervention, CI-Confidence Interval, ARR-Absolute risk reduction.

eFigure XII – Oral anticoagulation and Original Primary Outcomes of Individual Trials

Association of Oral Anticoagulation with Primary outcome

Study, Year	Int Event	Int Total	Control Event	Control Total	ARR (95% CI)	Weight	Odds Ratio (95% CI)
**Atrial fibrillation**							
AVERROES, 2011 [15]	51	2808	113	2791	2.23 [1.35 to 3.1]	7.03%	0.44 [0.31, 0.61]
BAFTA, 2007 [17]	76	488	100	485	5.04 [0.22 to 9.9]	7.10%	0.71 [0.51, 0.99]
WASPO, 2007 [16]	9	36	17	39	18.59 [-2.44 to 39.6]	2.19%	0.43 [0.16, 1.15]
Vemmos et al, 2006 [18]	0	16	2	15	12.68 [-6.84 to 32.2]	0.27%	0.16 [0.01, 3.71]
ACTIVE W, 2006 [19]	165	3371	234	3335	2.12 [0.99 to 3.2]	8.57%	0.68 [0.56, 0.84]
SPEAF (intermed), 2004 [20]	14	237	17	242	1.12 [-3.28 to 5.5]	3.36%	0.83 [0.40, 1.73]
PATAF-stratum 1, 1999 [21]	10	131	12	141	0.88 [-5.60 to 7.3]	2.62%	0.89 [0.37, 2.13]
AFASAK II, 1998 [22]	12	170	10	169	-1.14 [-6.38 to 4.1]	2.65%	1.21 [0.51, 2.88]
SPAF II (>75), 1994 [23]	29	197	27	188	-0.36 [-7.40 to 6.7]	4.56%	1.03 [0.58, 1.81]
SPAF II (<75), 1994 [23]	32	358	40	357	2.27 [-2.14 to 6.7]	5.29%	0.78 [0.48, 1.27]
EAFT, 1993 [24]	43	225	266	782	14.90 [8.79 to 21.0]	6.68%	0.46 [0.32, 0.66]
Veterans, 1992 [26]	4	260	19	265	5.63 [2.18 to 9.1]	1.86%	0.20 [0.07, 0.60]
BAATAF, 1992 [25]	2	212	13	208	5.31 [1.77 to 8.8]	1.08%	0.14 [0.03, 0.64]
SPAF (Group I), 1991 [27]	10	210	24	211	6.61 [1.45 to 11.8]	3.17%	0.39 [0.18, 0.84]
CAFA, 1991 [28]	8	187	11	191	1.48 [-2.92 to 5.9]	2.38%	0.73 [0.29, 1.86]
AFASAK, 1989 [29]	5	335	41	672	4.61 [2.38 to 6.8]	2.36%	0.23 [0.09, 0.60]
RE Model for Subgroup (Q = 30.24, df = 15, p = 0.01; I^2 = 49.2%)					3.1 [2 to 3.8]		0.58 [0.47, 0.72]
**Heart failure**							
COMPASS, 2019 [30]	24	236	29	240	1.91 [-3.73 to 7.6]	4.51%	0.82 [0.46, 1.46]
COMMANDER HF, 2018 [6]	626	2507	658	2515	1.19 [-1.22 to 3.6]	9.34%	0.94 [0.83, 1.07]
WARCEF, 2012 [31]	302	1142	320	1163	1.07 [-2.55 to 4.7]	8.80%	0.95 [0.79, 1.14]
WATCH, 2009 [4]	106	540	221	1047	1.48 [-2.68 to 5.6]	7.95%	0.91 [0.70, 1.18]
HELAS, 2006 [32]	19	92	24	105	2.20 [-9.32 to 13.7]	3.69%	0.88 [0.45, 1.73]
WASH, 2004 [5]	23	89	55	190	3.10 [-8.04 to 14.2]	4.55%	0.86 [0.48, 1.51]
RE Model for Subgroup (Q = 0.36, df = 5, p = 1.00; I^2 = 0.0%)					1.3 [-0.4 to 3]		0.93 [0.85, 1.02]
Heterogeneity: τ^2 = 0.07, χ^2 = 60.14, P = 0.00, I^2 = 66.5					2.5 [2 to 3.2]	100.00%	0.69 [0.59, 0.82]

Test for overall effect: z = -4.36, P = 0.00

P for interaction = 0.00

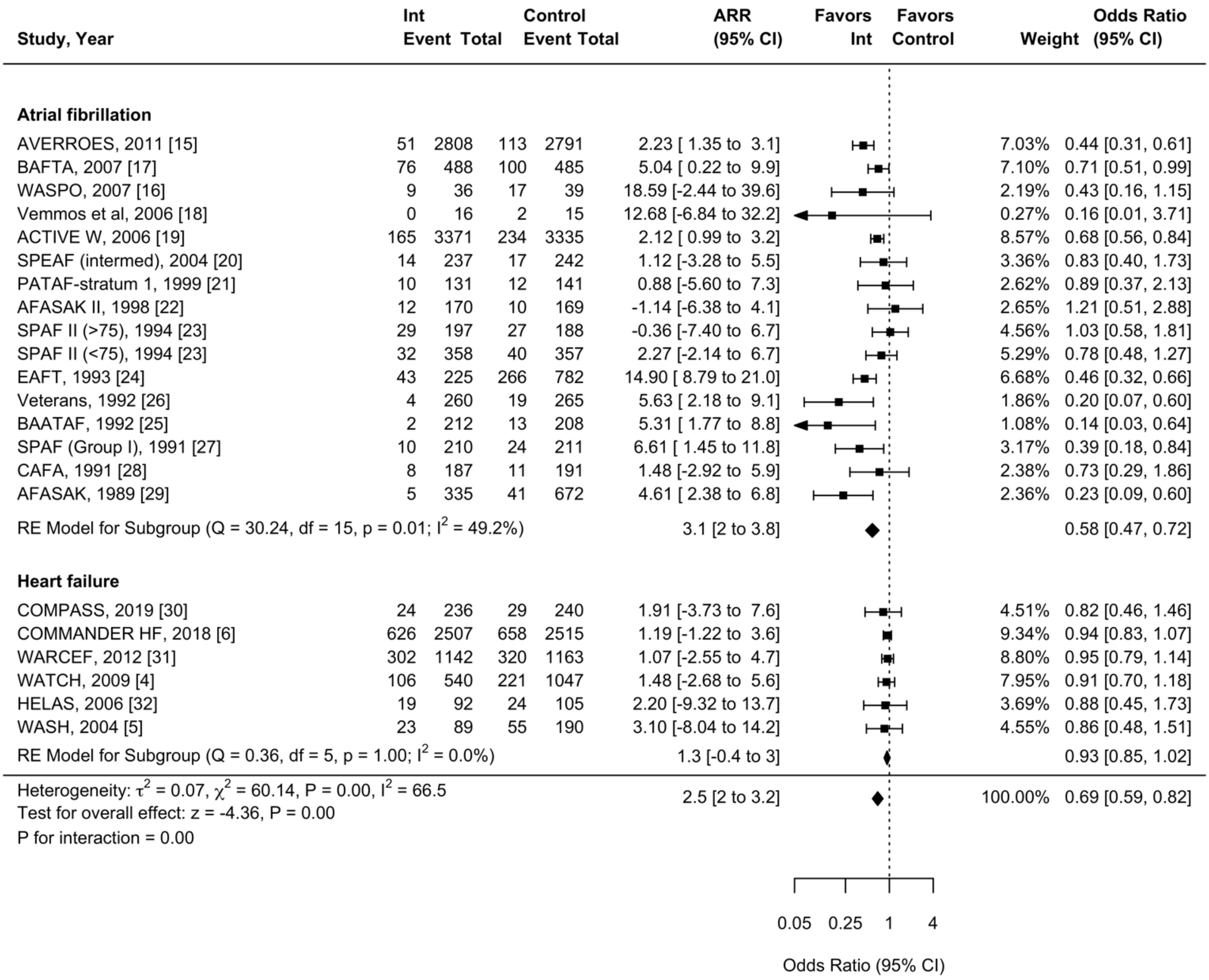

eFigure XII – Forest plot demonstrating the association of oral anticoagulant with original primary outcome reported within trials compared to control. The squares and bars represent the mean values and 95% confidence intervals of the effect sizes, while the size of the squares reflects the weight of the studies. The combined effects appear as diamonds and the vertical dashed line represents the line of no effect. Int-Intervention, CI-Confidence Interval, ARR-Absolute risk reduction.

eFigure XIII – Oral anticoagulation and All stroke - Sensitivity analyses

All Stroke Sensitivity analysis

Outcome	No. of studies	No. of patients	OAC	Control	I^2	Odds Ratio (95% CI)	P for interaction
**Low risk of bias**							
Atrial fibrillation	10	17017	224	535	31.9	0.53 [0.43, 0.67]	
Heart failure	5	9669	93	168	0	0.61 [0.47, 0.79]	
Overall	15	26686	317	703	16.5	0.55 [0.46, 0.65]	0.47
**Control - Aspirin**							
Atrial fibrillation	10	10824	165	451	36.6	0.53 [0.40, 0.70]	
Heart failure	5	4762	44	94	0	0.56 [0.39, 0.81]	
Overall	15	15586	209	545	21.8	0.53 [0.42, 0.66]	0.81
**Intervention - Warfarin**							
Atrial fibrillation	13	13254	189	487	34.8	0.52 [0.40, 0.68]	
Heart failure	4	4368	39	88	6.9	0.52 [0.32, 0.85]	
Overall	17	177622	228	575	22.2	0.52 [0.42, 0.65]	0.97
**INR target (2-3.5)**							
Atrial fibrillation	7	9222	118	194	0	0.58 [0.47, 0.71]	
Heart failure	4	4368	39	88	6.9	0.52 [0.32, 0.85]	
Overall	11	13590	157	282	0	0.58 [0.47, 0.71]	0.63

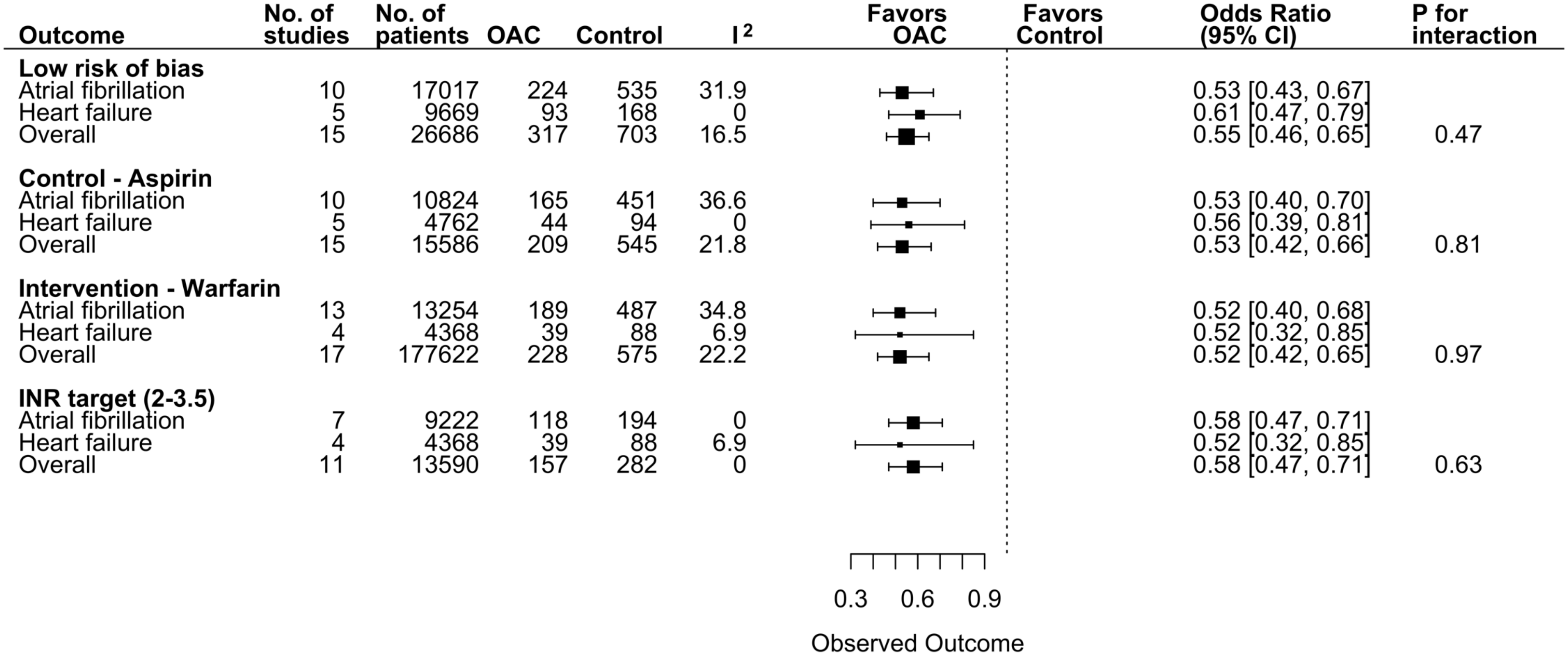

eFigure XIII – Combined Forest Plot showing sensitivity analysis for all stroke outcome: [1] Low risk of bias, [2] aspirin as control, [3] warfarin as intervention, [4] INR target (2-3.5).The analysis is divided by population group; overall, atrial fibrillation trials, heart failure trials. The squares and bars represent the mean values and 95% confidence intervals of the effect sizes, while the area of the squares reflects the weight of the studies. Int-Intervention, CI-Confidence Interval, ARR-Absolute risk reduction.

eFigure XIV – Oral anticoagulation and Ischaemic stroke - Sensitivity analyses

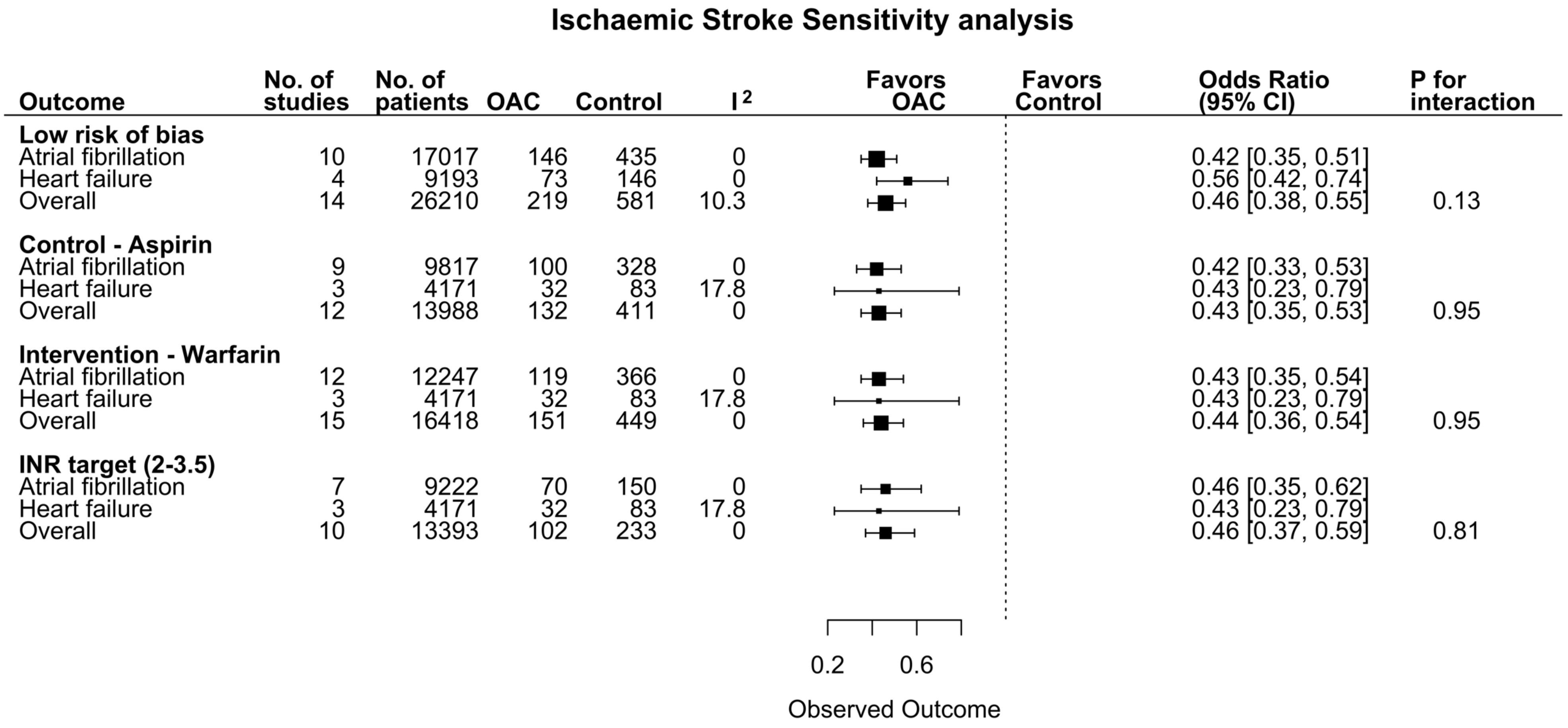

eFigure XIV – Combined Forest Plot showing sensitivity analysis for ischaemic stroke outcome: [1] Low risk of bias, [2] aspirin as control, [3] warfarin as intervention, [4] INR target (2-3.5).The analysis is divided by population group; overall, atrial fibrillation trials, heart failure trials. The squares and bars represent the mean values and 95% confidence intervals of the effect sizes, while the area of the squares reflects the weight of the studies. Int-Intervention, CI-Confidence Interval, ARR-Absolute risk reduction.

eFigure XV – Oral anticoagulation and Haemorrhagic stroke - Sensitivity analyses

Hemorrhagic Stroke Sensitivity Analysis

Outcome	No. of studies	No. of patients	OAC	Control	I^2	Odds Ratio [95% CI]	P for interaction
**Low risk of bias**							
Atrial fibrillation	9	15917	31	24	13.7	1.33 [0.70, 2.54]	
Heart failure	4	9193	11	10	7.9	1.20 [0.47, 3.07]	
Overall	13	25110	42	34	9.1	1.37 [0.81, 2.33]	0.86
**Control - Aspirin**							
Atrial fibrillation	8	8717	15	19	0	0.90 [0.46, 1.77]	
Heart failure	3	4171	5	2	0	2.41 [0.59, 9.88]	
Overall	11	12228	20	20	0	1.08 [0.59, 1.98]	0.22
**Intervention - Warfarin**							
Atrial fibrillation	10	10769	25	15	0	1.73 [0.91, 3.28]	
Heart failure	3	4171	5	2	0	2.41 [0.59, 9.88]	
Overall	13	14940	30	17	0	1.83 [1.02, 3.28]	0.68
**INR target (2-3.5)**							
Atrial fibrillation	6	8844	24	14	5	1.77 [0.96, 3.26]	
Heart failure	3	4171	5	2	0	2.41 [0.59, 9.88]	
Overall	9	13015	29	16	0	1.77 [0.96, 3.26]	0.62

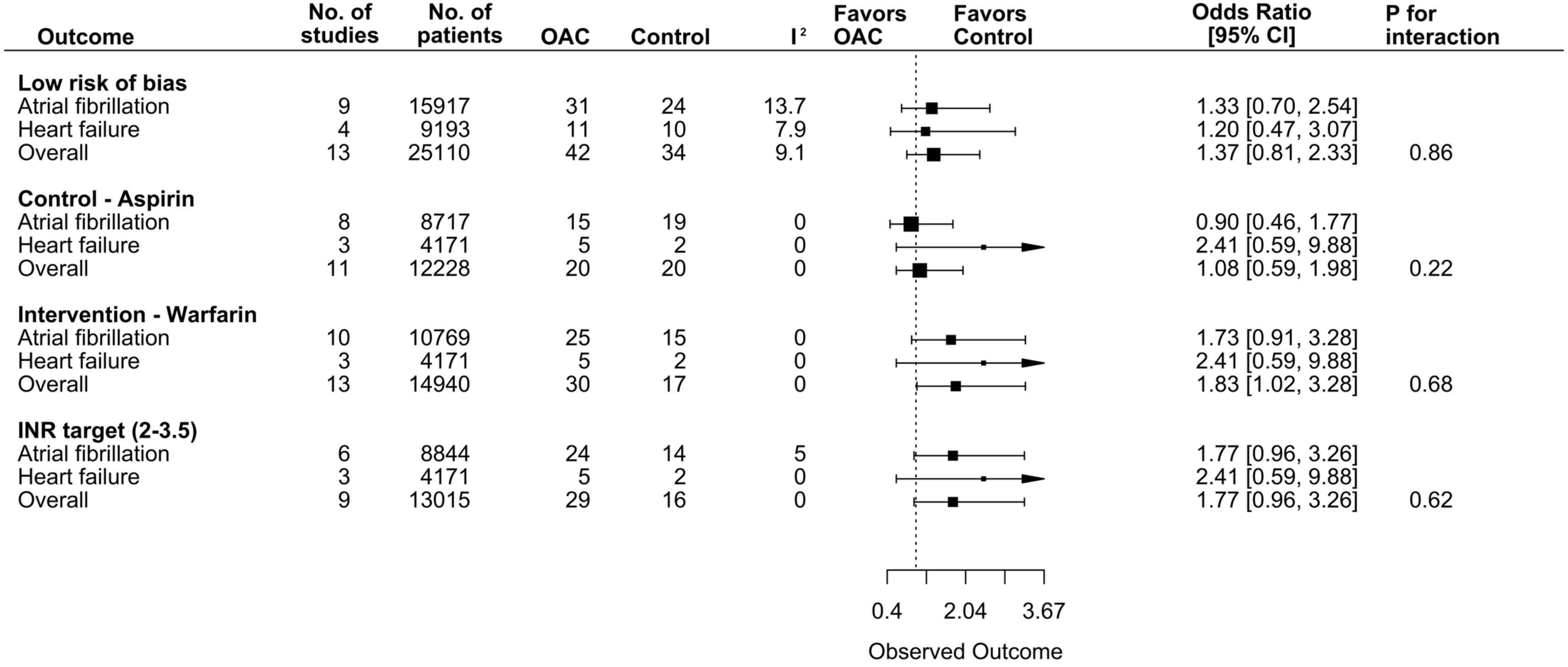

eFigure XV – Combined Forest Plot showing sensitivity analysis for hemorrhagic stroke outcome: [1] Low risk of bias, [2] aspirin as control, [3] warfarin as intervention, [4] INR target (2-3.5).The analysis is divided by population group; overall, atrial fibrillation trials, heart failure trials. The squares and bars represent the mean values and 95% confidence intervals of the effect sizes, while the area of the squares reflects the weight of the studies. Int-Intervention, CI-Confidence Interval, ARR-Absolute risk reduction.

eFigure XVI – Oral anticoagulation and All-cause mortality - Sensitivity analyses

All-cause Mortality Sensitivity analysis

Outcome	No. of studies	No. of patients	OAC	Control	I^2	Odds Ratio (95% CI)	P for interaction
**Low risk of bias**							
Atrial fibrillation	10	17017	530	736	4.5	0.87 [0.76, 0.99]	
Heart failure	5	9669	958	1090	0	0.99 [0.90, 1.09]	
Overall	15	266686	1488	1826	0	0.94 [0.87, 1.01]	0.11
**Control - Aspirin**							
Atrial fibrillation	9	9817	357	555	0	0.83 [0.72, 0.97]	
Heart failure	5	4762	423	543	0	1.01 [0.87, 1.16]	
Overall	14	14579	780	1098	6.7	0.91 [0.82, 1.02]	0.08
**Intervention - Warfarin**							
Atrial fibrillation	11	11827	429	604	0	0.90 [0.79, 1.04]	
Heart failure	5	4368	402	525	0	1.00 [0.86, 1.16]	
Overall	16	16195	831	1129	0	0.95 [0.86, 1.05]	0.33
**INR target (2-3.5)**							
Atrial fibrillation	7	9222	325	323	0	1.00 [0.85, 1.18]	
Heart failure	5	4368	402	525	0	1.00 [0.86, 1.16]	
Overall	12	13590	727	848	0	1.00 [0.90, 1.12]	0.96

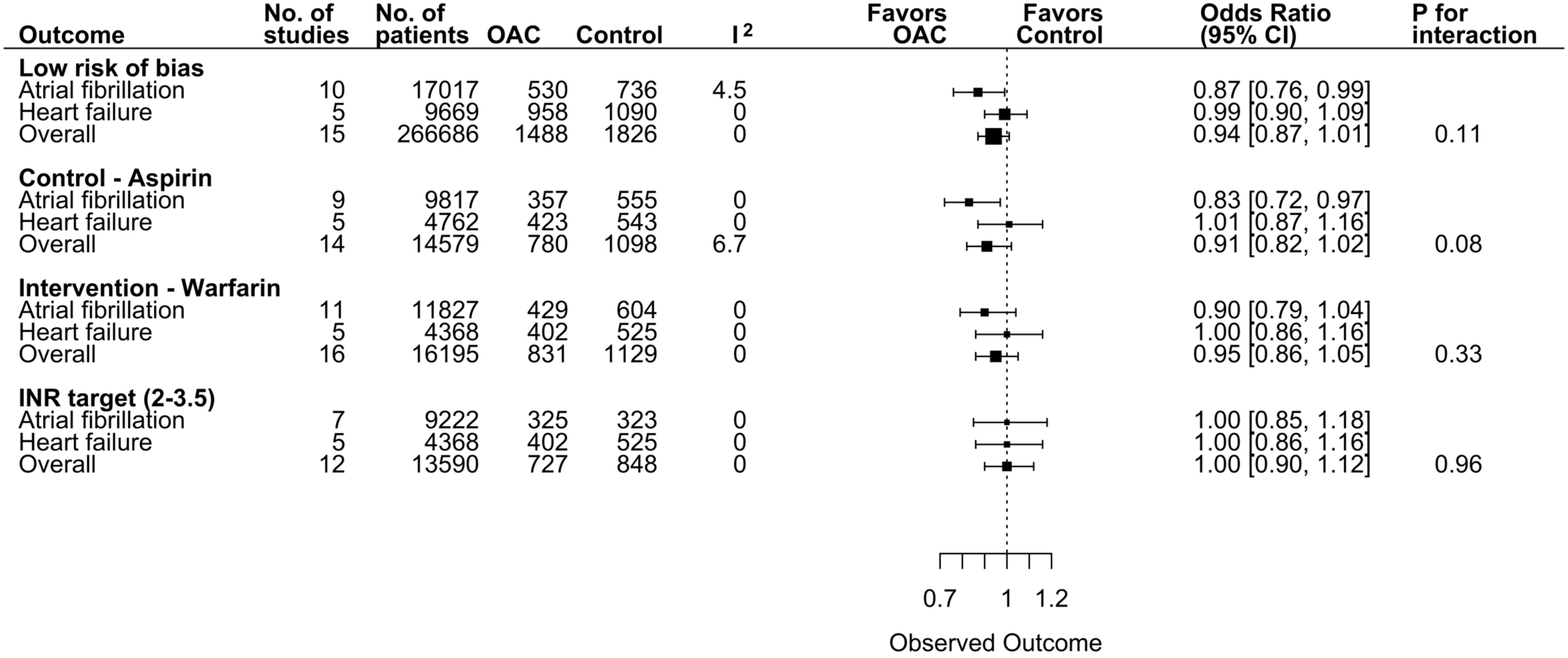

eFigure XVI – Combined forest plot reporting sensitivity analyses for the all-cause mortality outcome: [1] Low risk of bias, [2] control - aspirin, [3] intervention - warfarin, [4] INR target (2-3.5). The analysis is divided by population group; atrial fibrillation trials, heart failure trials, and overall. The squares and bars represent the mean values and 95% confidence intervals of the effect sizes, while the area of the squares reflects the weight of the studies. Int-Intervention, CI-Confidence Interval, ARR-Absolute risk reduction.

eFigure XVII – Composite MACE outcome (non-fatal MI, non-fatal stroke, all-cause mortality)

Association of Oral Anticoagulation with MACE Composite outcome

Study, Year	Int Event	Int Total	Control Event	Control Total	ARR (95% CI)	Weight	Odds Ratio (95% CI)
**Atrial fibrillation**							
AVERROES, 2011 [15]	157	2808	228	2791	2.6 [1.2 to 3.90]	11.57%	0.67 [0.54, 0.82]
BAFTA, 2007 [17]	126	488	147	485	4.5 [-1.1 to 10.13]	9.80%	0.80 [0.60, 1.06]
Vemmos et al, 2006 [18]	0	16	2	15	12.7 [-6.8 to 32.20]	0.22%	0.16 [0.01, 3.71]
ACTIVE W, 2006 [19]	255	3371	213	3335	-1.2 [-2.4 to 0.04]	12.13%	1.20 [0.99, 1.45]
SPEAF (intermed), 2004 [20]	24	237	25	242	0.2 [-5.2 to 5.63]	4.44%	0.98 [0.54, 1.77]
PATAF-stratum 1, 1999 [21]	16	131	22	141	3.4 [-4.8 to 11.59]	3.51%	0.75 [0.38, 1.51]
AFASAK II, 1998 [22]	30	170	24	169	-3.5 [-11.2 to 4.33]	4.50%	1.29 [0.72, 2.32]
EAFT, 1993 [24]	53	225	296	782	14.3 [7.8 to 20.80]	8.41%	0.51 [0.36, 0.71]
RE Model for Subgroup (Q = 30.50, df = 7, p = 0.00; I^2 = 73.8%)					1.4 [0.5 to 2.3]		0.82 [0.63, 1.07]
**Heart failure**							
COMPASS, 2019 [30]	30	236	34	240	1.4 [-4.7 to 7.58]	5.18%	0.88 [0.52, 1.50]
COMMANDER HF, 2018 [6]	626	2507	658	2515	1.2 [-1.2 to 3.61]	13.59%	0.94 [0.83, 1.07]
WARCEF, 2012 [31]	208	1142	232	1163	1.7 [-1.5 to 4.94]	11.63%	0.89 [0.73, 1.10]
WATCH, 2009 [4]	106	540	221	1047	1.5 [-2.7 to 5.64]	10.33%	0.91 [0.70, 1.18]
WASH, 2004 [5]	23	89	55	190	3.1 [-8.0 to 14.25]	4.68%	0.86 [0.48, 1.51]
RE Model for Subgroup (Q = 0.27, df = 4, p = 0.99; I^2 = 0.0%)					1.4 [-0.2 to 3.1]		0.92 [0.84, 1.01]
Heterogeneity: τ^2 = 0.04, χ^2 = 31.86, P = 0.00, I^2 = 65.1					1.4 [0.6 to 2.3]	100.00%	0.86 [0.74, 1.00]

Test for overall effect: z = -2.01, P = 0.04

P for interaction = 0.42

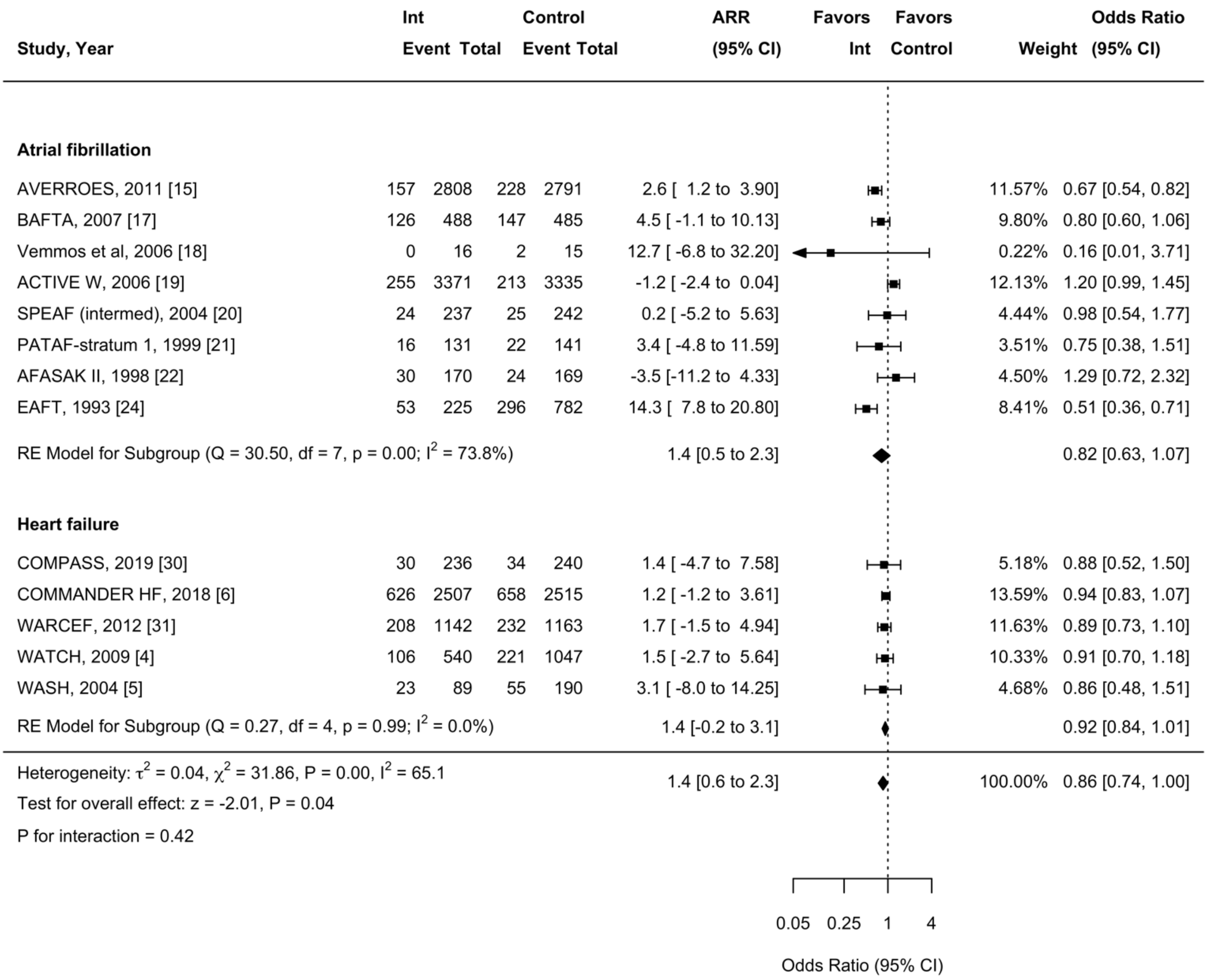

eFigure XVII – Forest plot demonstrating the effect of oral anticoagulant on MACE composite events compared to control. The squares and bars represent the mean values and 95% confidence intervals of the effect sizes, while the size of the squares reflects the weight of the studies. The combined effects appear as diamonds and the vertical dashed line represents the line of no effect. Int-Intervention, CI-Confidence Interval, ARR-Absolute risk reduction.

* Upper limit of confidence interval is 0.996 corrected to three decimal places.

eFigure XVIII – Composite of Non-fatal Cardiovascular events in HFrEF Trials

Primary composite outcome removing all-cause mortality

Study	Int Event	Total	Control Event	Total	Weight	Odds Ratio (95% CI)
Commander HF	149	2507	194	2515	66.14%	0.76 [0.61, 0.94]
HELAS(DCM)	5	38	5	44	1.85%	1.18 [0.31, 4.44]
HELAS(IHD)	5	54	5	61	1.92%	1.14 [0.31, 4.18]
WARCEF	34	1142	57	1163	17.26%	0.60 [0.39, 0.92]
WASH	1	89	11	190	0.76%	0.18 [0.02, 1.46]
WATCH	22	540	47	1047	12.08%	0.90 [0.54, 1.52]
Heterogeneity: $\tau^2 = 0.00$, $\chi^2 = 4.22$, P = 0.52, $I^2 = 0.0$<br>Test for overall effect: z = -3.20, P = 0.00					100.00%	0.75 [0.62, 0.89]

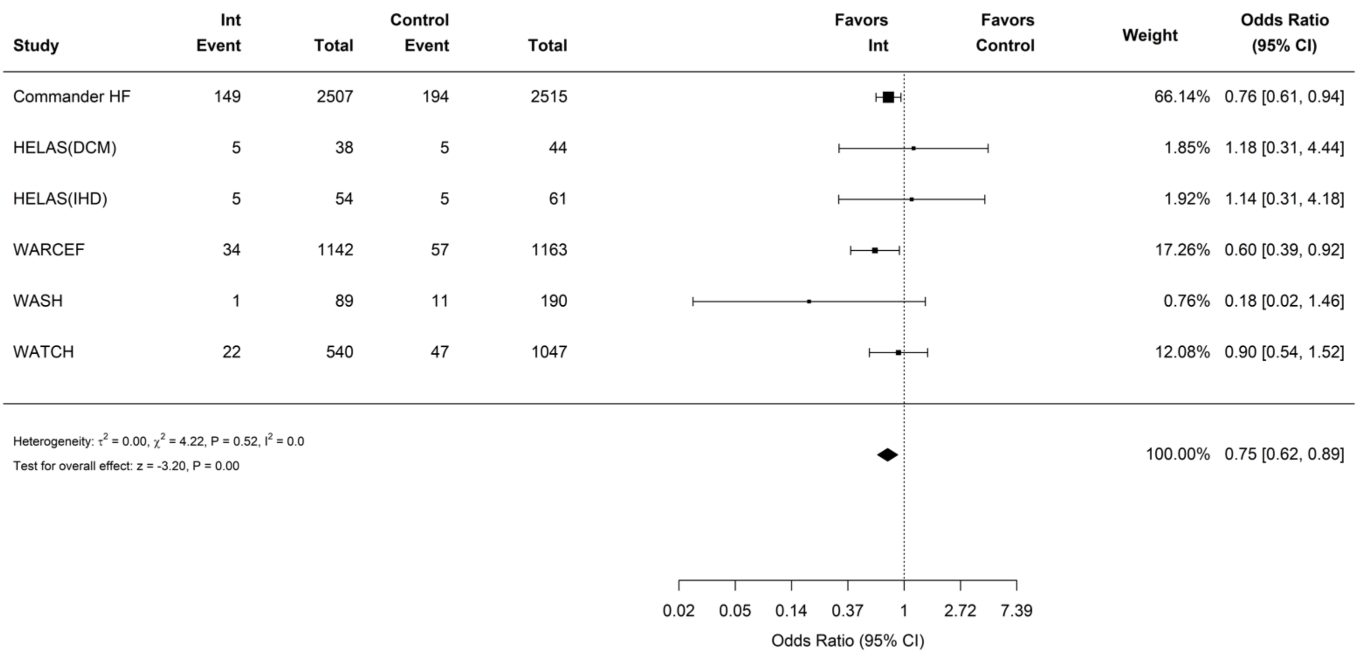

eFigure XVIII – Forest plot demonstrating the effect of oral anticoagulant on non-fatal cardiovascular events in HFrEF trials compared to control. The squares and bars represent the mean values and 95% confidence intervals of the effect sizes, while the size of the squares reflects the weight of the studies. The combined effect appear as a diamond and the vertical dashed line represents the line of no effect. Int-Intervention, CI-Confidence Interval.
